# Supplementary material for: Costing interventions in the field: preliminary cost estimates and lessons learned from an evaluation of community-wide mass drug administration for elimination of soil-transmitted helminths in the DeWorm3 trial
Source: BMJ Open. 2021 Jul 5;11(7):e049734. doi: 10.1136/bmjopen-2021-049734 (PMC8258667; doi:10.1136/bmjopen-2021-049734)
Supplement: Supplementary data [file bmjopen-2021-049734supp001.pdf]

Supplementary Information

Costing interventions in the field: preliminary cost estimates and lessons from an evaluation of community-wide MDA for elimination of STH

Galactionova K[1,2], Sahu M[1,2], Gideon SP[3], Kaliappan SP[3], Morozoff CE[4], Ajjampur SSR[3], Walson J[4], Means AR[4], Tediosi F[1,2]

[1] Swiss Tropical and Public Health Institute, Basel, Switzerland
[2] University of Basel, Basel, Switzerland
[3] Christian Medical College, Vellore, India
[4] University of Washington, Seattle, Washington, USA

Corresponding author:

Katya Galactionova
Socinstrasse 57,
4051 Basel,
Switzerland
e.galactionova@unibas.ch

Box S1. Key terms and definitions

Financial costs: These costs represent actual expenditure on goods and services purchased. Costs are described in terms of how much money has been paid for the resources used in the project or service.

Economic costs: Economic costs, on the other hand, include in addition to financial also costs forgone by using a resource in a particular way. These economic or opportunity costs recognize and value the cost of using resources, as these resources are then unavailable for productive use elsewhere.

Fixed costs: Also referred to as capital costs, indirect costs or overheads are expenses which do no depend with the quantity of output or services delivered by the project. These include rent, equipment lease payments, some wages and salaries.

Variable costs: Are costs that change in proportion to the output or services delivered by the projects. These include supplies, food, fee for service.

Resource category: In the Tool inputs required for implementation of STH MDA-related activities are broadly categorized into *Running costs* and *Activity costs*. *Running costs* refer to resources essential to setting up and running day-to-day operations of the trial. These inputs are employed in a range of trial activities (i.e. shared costs), are retained for the duration of the trial, and often represent a one-time investment (i.e. capital costs or start-up costs). *Activity costs*, on the other hand, refer to resources essential to implementation of specific activities, incremental to those identified under running costs. These inputs are acquired for a specific activity, are retained for the duration of the activity, and often represent recurrent purchases (i.e. require purchase each time the activity is implemented).

Activity: Throughout the trial a variety of activities will be implemented to plan for, deliver, and assess STH MDA. The Tool covers only activities implemented by the DeWorm3 trial team; these include implementation of MDA and related activities in the intervention arm of the trial and activities related to census, monitoring and evaluation, and surveillance in the control arm. Table1 above details the list of activities by implementing partner and trial arm.

Sub-activity: Each activity in turn is divided into sub-activities that represent specific steps or functions necessary to conclude the respective activity. For example “Stool sample collection” is a sub-activity within the “Cross-sectional survey” activity.

Cost classification: Within each sub-activity resources are further grouped into one of seven input classifications; the categories are defined in Table 2 below.

Resource line item: This is a single cost in the Tool. All line items will be associated with a description, cost classification, sub-activity, activity, and resource category.

Table S1. DeWorm3 trial activities

Activity	Study year (Y)	Tool	Sample	Primary focus	Contribution to economic analyses
Study area census	Y1	Survey CTO	Study area	Enumeration of residents in study area, socio-demographic information, GPS coordinates of dwellings	• Age<br>• School attendance<br>• Population size<br>• Occupation<br>• Asset ownership, dwelling<br>• WASH<br>• Catchment area<br>• Distance to health facility
School facility surveys	Y1-Y5	Survey CTO	Study area	WASH, school enrollment and attendance rates of children	• MDA conducted at school<br>• Time teacher spend helping with the drug delivery<br>• Teacher trained for school de-worming in past 6 months
Cross-sectional prevalence surveys	Y1, Y4, Y5	Kato-Katz, qPCR	500 (1000 for end-line) individuals per cluster	STH prevalence and intensity, DNA	• Deworming in past year<br>• Deworming source<br>• WASH
Longitudinal prevalence surveys	Y1-Y5	Kato-Katz, qPCR	150 individuals per cluster	STH prevalence, intensity, re-infection, DNA	• Time (minutes) CDD spent at the house on the treatment day<br>• Time spent participate in the treatment day, including time waiting for CDD
cMDA	Y1-Y3	Survey CTO	Intervention clusters	MDA coverage	
Coverage surveys	Y1-Y3	Urine test	50 households per cluster	MDA coverage	• Health workers bring medicines or other treatments by the house (last month)<br>• Health worker left tables for intake later<br>• Number of tablets swallowed<br>• Tablets swallowed while the health worker present<br>• Awareness of drug distribution visit<br>• Change to routine to participate in the treatment<br>• Time (minutes) CDD spent at the house on the treatment day<br>• Time spent participate in the treatment day, including time waiting for CDD

For further details on the study protocol refer to Asbjornsdottir et al PLOS 2018

Table S2 Activity Table: cMDA-related activities at the India site in the first trial cycle

Nr	Activity	Sub-activity	Trial arm	Implementation	Resource use
1	Drug procurement and supply chain	Procurement	*Both*	*- Drugs for both arms procured centrally by MoH as part of NTD program procurement<br>- DW3 is part of the national bulk purchase of donated drugs through WHO*	*- GSK donating 200,000 doses to the national NTD program for the trial<br>- Annual procurement<br>- Albendazole testing*
		Storage	*Both*	*DW3 trial drugs are stored at central CMC office with A/C (not paid by DW3)*	*Space requirements for 1 round MDA: 1-2 cupboards to keep 25 medium size boxes of tablets (6ft * 4 ft); total space needed for all rounds MDA: 10ft*10ft*
		Transportation	*Both*	*Grants manager travelled to Mumbai to the Government Medical Depot to take the drugs and arrange courier for shipment to DW3 office in Vellore. From Vellore, drugs were taken to sites using DW3 project vehicle.*	*- Grants manager flight to/from Mumbai, per-diems, accommodation<br>- Courier charges Mumbai to Vellore<br>- Fuel Vellore to field sites*
2	Community-wide MDA round 1 *(implemented as mop-up 1 week after SBD)*	Community sensitization	*Int*	*- Implemented right after NDD<br>- Central DW3 team adapts government developed materials to the trial<br>- State health authorities and local social scientists provide input to IEC adaptation through meetings at central level<br>- ASHA FOs and FSs workers organize and run village meetings*	
		Recruitment of CDDs	*Int*	*- 2 days<br>- 164 total CDDs recruited: 32 were already available, 132 were scouted*	*-Staff time:<br>TC: 1 day<br>FM: 2 days<br>FS (11): 4 days<br>FOs (all): 2 days<br>- Transportation allowance: field staff 3Rs/km<br>-Hired vehicle to transport recruiters*
		Training and Piloting	*Int*	*- CDDs 3 days:<br>CDDS/ASHA workers are trained by DW3 Medical Officer at cluster level.<br>- FOs FS 4 days:<br>FOs (109) and FS (11) trained on forms by TC, DM, TL<br>- AE/SAE training for Medical Team 1 day:<br>MO gave training to 2 DW3 study nurses*	*- CDD/ASHAs received travel reimbursement (100 rupees) and refreshments<br>-Travel reimbursement and refreshments for CDD/ASHA workers<br>- No refreshments provided, only regular transport allowance for FOs, FS<br>- No transport allowance for AE/SAE trainings<br>-Fuel for vehicle to transport TC, DM, TL to field (4 days)*
		MDA	*Int*	*- DW3 FS supervise MDA<br>- MDA delivered by CDDs accompanied by FOs, all by foot<br>- 2 MOs and 2 nurses support in the field with AE<br>- 164 CDDs/ASHA workers total, working on average 5 days each for a total of 26 days worked.*	*-108 FOs<br>-1 CDD at a time per FW<br>-Village health nurse hired for 15 days to sensitize, administer and mop-up MDA<br>- CDDs/ASHA workers are paid an honorarium (200 Rs/day) + mobile allowance (50 Rs/round) for a total of 173,400Rs<br>- 44 VHNs , SHNs, CHNs paid 500 Rs/round for a total of 22,000Rs<br>- Office supply costs including printing of logs<br>- Job aids, bags, posters, banners, flip charts*

		Post-MDA coverage survey	*Int*	*- Includes 1 day of training for FOs by TC and DM<br>- Data processing done centrally by local data team*	*- Staff time: DM,TC,FOs<br>- Office supply costs including printing of logs*
		Mop-up MDA	*Int*	*- FOs and FS did mop-up and a few CDDs/ASHA*	*No allowances given to CDDs/ASHA*
3	Drug procurement and supply chain	Procurement	*Int*	*- Drugs for both arms procured centrally by MoH as part of NTD program procurement<br>- DW3 is part of the national bulk indent of donated drugs through WHO*	*-GSK donating 200,000 doses to the national NTD program for the trial<br>- Albendazole quality control testing*
		Storage	*Int*	*DW3 trial drugs are stored at central CMC office with A/C (not paid by DW3)*	*Space requirements for 1 round MDA: 1-2 cupboards to keep 25 medium size boxes of tablets (6ft * 4 ft); total space needed for all rounds MDA: 10ft*10ft*
		Transportation	*Int*	*Grants Manager travelled to Mumbai to the Government Medical Depot to collect the drugs and arrange courier for shipment to DW3 office in Vellore. From Vellore, drugs were taken to sites using DW3 project vehicle.*	*- Grants Manager flight to/from Mumbai, per-diems, accommodation<br>- Courier charges Mumbai to Vellore<br>- Fuel Vellore to field sites*
4	Community-wide MDA round 2	Community sensitization	*Both*	*- CAB meetings 1 day:<br>TC, PI, FM, MO, IS (4), FS (8), 12 CAB members<br>- Community sensitization meetings 11 days: FM and 3 FS and 5 FO went to 82 villages conducted 82 meetings (5 persons per meeting)<br>- Joined meetings organized by Grama Sabha to sensitize village heads and local community leaders<br>- FS/FO travel by personal bikes*	*- Staff time: PI,TC,MO,FM, FS<br>- Food expense for community-level CAB meetings<br>- BMOs 1000Rs honorarium, others (schoolteachers, block superintendent, opinion leaders etc) 500Rs, Food 237 Rs/person<br>- FS/FOs receive transport allowance 3 Rs per km<br>- Fuel for DW3 project staff (CMC vehicle)*
		Training and Piloting	*Int*	*- CDDs/ASHA 2 days: CDDS/ASHA workers are trained by DW3 MO at cluster level<br>- 4 days: FOs (60) and FS (10) trained on forms by TC, DM, TL.<br>-AE/SAE training for Medical Team 1 day: MO gave training to 2 DW3 study nurses*	*- CDD/ASHAs received travel reimbursement (100 rupees) and refreshments<br>- No refreshments provided, only regular transport allowance for FO, FS trainings<br>- No transport allowance for AE trainings<br>-Fuel for vehicle to transport TC, DM, TL to field (4 days)*
		MDA	*Int*	*-DW3 field supervisors supervise MDA<br>-MDA delivered by CDDs accompanied by FO (70), all on foot<br>- 2 Medical officers and 2 nurses support in the field with adverse event<br>-114 CDDs/ASHA workers total, working on average 5 days each for a total of 12 days worked.*	*- CDDs/ASHA workers are paid an honorarium (200 Rs/day) + mobile allowance (50 Rs/round) for a total of Rs188,650<br>- Office supply costs including printing of logs<br>- Job aids, bags, posters, banners, flip charts*
		Post-MDA coverage survey	*Both*	*- Includes 1 day of training for FOs by TC and DM<br>- Data processing done centrally and by local data team*	*- Staff time: DM,TC, FOs, DM<br>- Printing charges<br>- Office supply costs including printing of logs*
		Mop-up MDA	*Int*	*- FOs and FS did mop-up (and a few CDDs/ASHA)*	*No allowances given to CDDs/ ASHA workers*

Int= Intervention arm; MoH= Ministry of Health; MoE= Ministry of Education; CMC= Christian Medical College, Vellore; TC= trial coordinator; FM= field manager; FS= field supervisor; FO= field officer; DM= data manager

Table S3 Cost classification of inputs in the DeWorm3 costing tool

No	Input classification	Define	Examples
1	Drugs	Anti-helminthic medication distributed during MDA campaigns and used to treat adults with moderate to high intensity infections	Albendazole
2	Wages and per-diems	Fixed regular payment earned for work or services, per-diem allowances, and any other monetary awards paid to personnel involved in the trial	Per-diems
3	Vehicles and overheads	Means of transportation, including hire and transportation allowance, vehicle maintenance, fuel	Transportation allowance, fuel allowance
4	Equipment and overheads	Supplies and tools that last more than a year and have a unit cost at or above 100 USD; including maintenance and overheads	Mobile phones, Microscope, Equipment maintenance
4	Buildings and overheads	Facilities occupied by DeWorm3 trial and supporting teams	Rent payment, utilities incurred at DeWorm3 site offices
5	Materials and supplies	Commodities required to support an operation or activity	SIM cards, office supplies
6	Other	All other inputs outside of the either of the above categories	Consumables (i.e. food and drinks)

Galactionova K, *et al. BMJ Open* 2021; 11:e049734. doi: 10.1136/bmjopen-2021-049734

Table S4 Allocation of resource line items to trial activities and categorization of resource line-items into cMDA and research-related and allocation rules of management overheads to trial activities

Resource category	Resource line items	Activity	Activity allocation rule	cMDA	Research
Staff wages and per-diems	Trial PI and core trial staff	Program management	Permanent staff, direct allocation based on % time working on trial	Yes	Yes
	Field staff	Activity	Temporary staff, direct allocation based on number of days supporting activity	Yes	Yes
	Laboratory staff	Prevalence surveys	Permanent staff, direct allocation based on % time working on trial; temporary staff, direct allocation based on number of days supporting activity	No	Yes
	Data staff	Program management	100%	No	Yes
Vehicles and overheads	Vehicles owned	Program management	100%	Yes	Yes
	Vehicles rented	Program management	100%	Yes	Yes
	Transportation related expenses of field and other project staff	Activity	Direct allocation based on number of days supporting activity	Yes	Yes
Equipment and overheads	Office equipment	Program management	100%	Yes	Yes
	Mobile phones	Census	100%	No	Yes
	Field worker kits	Activity	100%	Yes	Yes
	Laboratory equipment	Prevalence survey	100%	No	Yes
Buildings and overheads	Central	Program management	100%	Yes	Yes
	Field offices	Program management	100%	Yes	Yes
Materials and supplies	Stationaries and other materials	Activity	Direct allocation based on number of days supporting activity	Yes	Yes
Other	Trial insurance	Program management	100%	No	Yes

Table S5 Average annual financial costs of STH cMDA* implementation in India DeWorm3 trial site, USD 2018

Sub-activity	Start-up**		Program management		Drug testing and distribution to sites		CDD recruitment		Community sensitization		Training		cMDA		Coverage survey		Mop-up	
Cost category	US$	%	US$	%	US$	%	US$	%	US$	%	US$	%	US$	%	US$	%	US$	%
Wages and per-diems***	2,027	94.3	26,520	63.4	0	0.0	1,199	57.2	11,283	50.7	4,322	55.1	18,666	66.4	3,232	58.1	2,388	57.8
Buildings and overheads	80	3.7	2,139	5.1	0	0.0	0	0.0	0	0.0	0	0.0	0	0.0	0	0.0	0	0.0
Equipment and overheads	0	0.0	857	2.0	0	0.0	0	0.0	7	0.0	0	0.0	0	0.0	0	0.0	0	0.0
Transportation	22	1.0	7,562	18.1	553	87.0	858	41.0	7,590	34.1	3,360	42.8	7,709	27.4	2,215	39.8	1,665	40.3
Communication	7	0.3	3,378	8.1	0	0.0	0	0.0	0	0.0	0	0.0	205	0.7	0	0.0	0	0.0
Materials and supplies	11	0.5	1,384	3.3	0	0.0	38	1.8	3,078	13.8	135	1.7	395	1.4	120	2.2	75	1.8
Other	2	0.1	0	0.0	83	13.0	0	0.0	309	1.4	26	0.3	1,126	4.0	0	0.0	0	0.0
Total	2,149	100.0	41,840	100.0	636	100.0	2,095	100.0	22,267	100.0	7,843	100.0	28,101	100.0	5,567	100.0	4,128	100.0

*cMDA was implemented as a mop-up following NDD campaign that targeted school-aged children; ** Start-up activities annualized over the duration of the trial (5 years). *** Wages and per-diems include project staff, field workers, and incentives to CDDs. Costs were converted to USD using average annual exchange rate over the study period (1 INR=0.01462 USD) [26]. STH= Soil-Transmitted Helminth infections; cMDA= community-wide Mass Drug Administration; CDD= Community Drug Distributor.

Table S6 First year financial costs of STH cMDA* implementation in India DeWorm3 trial site, USD 2018: adjusted base rate

	Total (US$)			Average cost Profile (%)	Average cost per person treated per round (US$)	Average cost per Capita per round (US$)
MDA round	1	2	1+2	1+2	1+2	1+2
**TOTAL**	62,819	57,672	120,491	100.0	1.20	0.88
**TOTAL incremental****	4,829	6,577	11,406	9.5	0.11	0.08
Start-up activities***	1,094	1,055	2,149	1.8	0.02	0.02
Program management	21,300	20,540	41,840	34.7	0.42	0.31
Drug testing and distribution to site	304	331	636	0.5	0.01	0.00
CDD recruitment	1,160	1,119	2,279	1.9	0.02	0.02
Community sensitization	13,972	10,235	24,207	20.1	0.24	0.18
CDD bags and job aids	555	428	983	0.8	0.01	0.01
Banners, posters	969	969	1,937	1.6	0.02	0.01
Training	4,826	3,222	8,048	6.7	0.08	0.06
Travel allowance for CDDs	240	170	409	0.3	0.00	0.00
Refreshments for CDDs	10	17	26	0.0	0.00	0.00
cMDA	14,495	16,065	30,560	25.4	0.30	0.22
CDD incentives	2,632	4,577	7,210	6	0.07	0.05
CDD mobile allowance	120	85	205	0.2	0.00	0.00
Coverage survey	3,404	2,803	6,207	5.2	0.06	0.05
Mop-up	2,262	2,302	4,564	3.8	0.05	0.03

*cMDA was implemented as a mop-up following NDD campaign that targeted school-aged children; **Incremental costs represent a subset of rows highlighted in *cursive*, see text for details. *** Start-up activities annualized over the duration of the trial (5 years). Grey shaded rows are a subset of the higher level activity grouping. Total number of people treated in first round of cMDA (cMDA1) was 51’320 (site total population 68’442); in second round (cMDA2) – 49’488 (site total population 68’460); total treated over the two rounds (cMDA1+cMDA2) was 100'808 (total population 136'902). Costs were converted to USD using average annual exchange rate over the study period (1 INR=0.01462 USD) [27]. STH= Soil-Transmitted Helminth infections; cMDA= community-wide Mass Drug Administration; CDD= Community Drug Distributor.

File 1. DeWorm3 Costing Tool data collection templates

Module1: Planning

A1.1 Planning

	Number of days						
Description	Recruitment	Procurement	Planning trial activities	Developing IEC materials	Developing training materials	Remarks	Source
I	III	IV	V	VI	VII	VIII	IX

A1.2 Pre-trial sensitization

A1.2.1 Pre-trial sensitization meetings

Level of meeting	Location	Type of meeting space	Length of meeting (days)	Number of meetings	Date(s)
National					
State					
District					
Block					
Public health center					
Health sub center					
Cluster					

Galactionova K, *et al. BMJ Open* 2021; 11:e049734. doi: 10.1136/bmjopen-2021-049734

A1.2.2 Pre-trial sensitization meetings attendees

	Sensitization meeting							
Description	Level of meeting	Trial staff (Yes/No)	Transportation allowance per meeting (LCU)	Daily lodging allowance (LCU)	Daily food allowance (LCU)	Other allowance (LCU)	Remarks	Source
I	II	III	IV	V	VI	VII	VIII	IX

A1.2.3 Pre-trial sensitization meetings attendees

	Sensitization meeting							
Description	Level of meeting	Trial staff (Yes/No)	Transportation allowance per meeting (LCU)	Daily lodging allowance (LCU)	Daily food allowance (LCU)	Other allowance (LCU)	Remarks	Source
I	II	III	IV	V	VI	VII	VIII	IX

Module2: Shared resources and program overheads

P2.1 Wages and per-diems

Description	Employed by trial (Yes/No)	Working on trial (%)	Number of staff units (units)	Monthly wage (LCU)	Other monthly benefits (LCU)	Daily per-diem rate (LCU)	Remarks	Source
I	II	III	XVI	XVII	XVIII	XIX	XX	XXI

P2.2 Vehicles and overheads

Description	Purchased by trial (Yes/ No)	Used by trial (%)	Number of vehicles (units)	Make	Year of productio n	Year of purchas e	Price (LCU)	Average annual overhead costs (LCU)	Average annual maintena nce costs (LCU)	Remarks	Source
I	II	III	IV	V	VI	VII	VIII	IX	X	XI	XII

Galactionova K, *et al. BMJ Open* 2021; 11:e049734. doi: 10.1136/bmjopen-2021-049734

P2.2.1 Reference fuel prices

Fuel	Price units	Price per unit (LCU)	Km per L	L per km	Remarks

P2.2.2 Reference distances

Location	Distance units	Distance (one way)

P2.2.3 Other transportation related expenses

Description	Monthly travel expenditures (LCU)										
	Month, year	Month, year	Month, year	Month, year	Month, year	Month, year	Month, year	Month, year	Month, year	Month, year	Month, year

P2.3 Equipment and overheads

Description	Purchased by DeWorm3 (Yes/ No)	Used by DeWorm3 (%)	Number of units (units)	Make	Year of production	Year of purchase	Total Price (LCU)	Import duties and other charges (LCU)	Average annual overhead costs (LCU)	Average annual maintenance costs (LCU)	Remarks	Source
I	III	IV	V	VI	VII	VIII	IX	X	XI	XII	XIII	XIV

P2.3.1 Reference office furnishings prices and calculations

Furnishings	Unit price (LCU)	Number of units (units)	Total (LCU)

P2.4 Buildings and overheads

Description	Rented by DeWorm3 (Yes/ No)	Used by DeWorm3 (%)	Number of facilities (units)	Monthly rent per sq foot (LCU)	Size (sq feet)	Average monthly overheads (LCU)	Total including monthly overheads (LCU)	Remarks	Source
I	III	IV	V	VI	VII	VIII	IX	X	XI

P2.5 Materials and supplies

Description	Number of units (units)	Rate per unit (LCU)	Rate units	Remarks	Source
I	II	III	IV	V	VI

P2.5.1 Other materials and supplies

Description	Monthly materials and supplies expenditures (LCU)										
	Month, year	Month, year	Month, year	Month, year	Month, year	Month, year	Month, year	Month, year	Month, year	Month, year	Month, year

P2.6 Other

Description	Purchased by DeWorm3 (Yes/ No)	Used by DeWorm3 (%)	Number of units (units)	Rate per unit (LCU)	Rate units	Other charges (LCU)	Total Charges (LCU)	Remarks	Source
I	II	III	*IV*	V	VI	VII	VIII	IX	X

Module3: Activity

A3.1 Sub-activity

Cost classification	Description	Number of units (units)	Units	Insurance (LCU)	Freight (LCU)	Travel charges (LCU)	Handling fee (LCU)	Total (LCU)	Remarks	Source
I	II	III	IV	V	VI	VII	VIII	IX	X	XI

Stata code for processing data entries collated with the templates to derive intervention costs can be made available on request from authors.

Galactionova K, *et al. BMJ Open* 2021; 11:e049734. doi: 10.1136/bmjopen-2021-049734
