# Supplementary material for: Costing interventions in the field: preliminary cost estimates and lessons learned from an evaluation of community-wide mass drug administration for elimination of soil-transmitted helminths in the DeWorm3 trial
Source: BMJ Open. 2021 Jul 5;11(7):e049734. doi: 10.1136/bmjopen-2021-049734 (PMC8258667; doi:10.1136/bmjopen-2021-049734)
Supplement: Author's manuscript [file bmjopen-2021-049734.draft_revisions.pdf]

BMJ Open

BMJ Open is committed to open peer review. As part of this commitment we make the peer review history of every article we publish publicly available.

When an article is published we post the peer reviewers' comments and the authors' responses online. We also post the versions of the paper that were used during peer review. These are the versions that the peer review comments apply to.

The versions of the paper that follow are the versions that were submitted during the peer review process. They are not the versions of record or the final published versions. They should not be cited or distributed as the published version of this manuscript.

BMJ Open is an open access journal and the full, final, typeset and author-corrected version of record of the manuscript is available on our site with no access controls, subscription charges or pay-per-view fees (http://bmjopen.bmj.com).

If you have any questions on BMJ Open's open peer review process please email info.bmjopen@bmj.com

BMJ Open

Costing interventions in the field: preliminary cost estimates and lessons from an evaluation of community-wide Mass Drug Administration for elimination of Soil-Transmitted Helminths in the DeWorm3 trial

Journal:	*BMJ Open*
Manuscript ID	bmjopen-2021-049734
Article Type:	Original research
Date Submitted by the Author:	31-Jan-2021

Complete List of Authors:	Galactionova, Katya; Swiss Tropical and Public Health Institute; University of Basel<br>Sahu, Maitreyi; Swiss Tropical and Public Health Institute; University of Basel<br>Gideon, Samuel; Christian Medical College Vellore<br>PUTHUPALAYAM KALIAPPAN, SARAVANAKUMAR; Christian Medical College Vellore<br>Morozoff, Chloe; University of Washington<br>Ajjampur, Sitara; Christian Medical College Vellore<br>Walson, Judd; University of Washington<br>Rubin Means, Arianna; University of Washington<br>Tediosi, Fabrizio; Swiss Tropical and Public Health Institute; University of Basel

Keywords:	HEALTH ECONOMICS, Health policy < HEALTH SERVICES ADMINISTRATION & MANAGEMENT, Protocols & guidelines < HEALTH SERVICES ADMINISTRATION & MANAGEMENT, Infection control < INFECTIOUS DISEASES, Public health < INFECTIOUS DISEASES, INFECTIOUS DISEASES

SCHOLARONE™
Manuscripts

I, the Submitting Author has the right to grant and does grant on behalf of all authors of the Work (as defined in the below author licence), an exclusive licence and/or a non-exclusive licence for contributions from authors who are: i) UK Crown employees; ii) where BMJ has agreed a CC-BY licence shall apply, and/or iii) in accordance with the terms applicable for US Federal Government officers or employees acting as part of their official duties; on a worldwide, perpetual, irrevocable, royalty-free basis to BMJ Publishing Group Ltd ("BMJ") its licensees and where the relevant Journal is co-owned by BMJ to the co-owners of the Journal, to publish the Work in this journal and any other BMJ products and to exploit all rights, as set out in our licence.

The Submitting Author accepts and understands that any supply made under these terms is made by BMJ to the Submitting Author unless you are acting as an employee on behalf of your employer or a postgraduate student of an affiliated institution which is paying any applicable article publishing charge ("APC") for Open Access articles. Where the Submitting Author wishes to make the Work available on an Open Access basis (and intends to pay the relevant APC), the terms of reuse of such Open Access shall be governed by a Creative Commons licence – details of these licences and which Creative Commons licence will apply to this Work are set out in our licence referred to above.

Other than as permitted in any relevant BMJ Author's Self Archiving Policies, I confirm this Work has not been accepted for publication elsewhere, is not being considered for publication elsewhere and does not duplicate material already published. I confirm all authors consent to publication of this Work and authorise the granting of this licence.

For peer review only

Costing interventions in the field: preliminary cost estimates and lessons from an evaluation of community-wide Mass Drug Administration for elimination of Soil-Transmitted Helminths in the DeWorm3 trial

Galactionova K[1,2], Sahu M[1,2], Gideon SP[3], Kaliappan SP[3], Morozoff C[4], Ajjampur SSR[3], Walson J[4], Means AR[4], Tediosi F[1,2]

[1] Swiss Tropical and Public Health Institute, Basel, Switzerland
[2] University of Basel, Basel, Switzerland
[3] Christian Medical College, Vellore, India
[4] University of Washington, Seattle, Washington, USA

Corresponding author:

Katya Galactionova
Socinstrasse 57,
4051 Basel,
Switzerland
e.galactionova@unibas.ch

Word count: 5560

For peer review only

For peer review only - http://bmjopen.bmj.com/site/about/guidelines.xhtml

Keywords:

costs, cost data collection, field trial, Soil-Transmitted Helminths, community-wide MDA

For peer review only

Author Information:

Katya Galactionova	e.galactionova@unibas.ch
Mita Sahu	msahu@uw.edu
Samuel Paul Gideon	sampaulgide@gmail.com
Saravanakumar Puthupalayam Kaliappan	epipks@gmail.com
Chloe Morozoff	chloemor@uw.edu
Sitara Swarna Rao Ajjampur	sitararao@cmcvellore.ac.in
Judd Walson	walson@uw.edu
Arianna Means	aerubin@uw.edu
Fabrizio Tediosi	fabrizio.tediosi@swisstph.ch

For peer review only

Abstract

Objective: To develop and apply a strategy for collecting cost data alongside field activities in a multi-country trial of community-wide MDA for elimination of helminth infections.

Design: Cluster-Randomized Field Trial.

Setting: Tamil Nadu State, India.

Participants: Adults and children in communities targeted by the national deworming campaign.

Primary and secondary outcome measures: Incremental cost of extending deworming of school-aged children to the whole community.

Results: Central to the methodology is delineation of responsibilities for data collection and economic valuation. On-site staff, guided by instruments and technical support from an external economics team, led cost data collection in parallel with trial activities. Resources that contributed to multiple activities were identified and assessed at the beginning of the trial. Thus for each activity, only expenditures additional to program overheads and capital were recorded, minimizing effort and facilitating allocation. The economics team triangulated the data, allocated costs of shared resources and valued capital and recurrent items to derive an estimate of MDA costs. The methodology produced costs that aligned well with the literature. In India, in the first year of the trial, average financial cost was estimated at 1.14 (USD, 2018) per person treated per round. With drugs donated by GSK, wages and incentives to field staff accounted for over 70% of total cMDA costs. The data allowed consideration of incremental costs also under routine rather than trial implementation. The hypothesized at-scale scenario yielded a substantially lower cost estimate of 0.11 USD per person treated per round.

Conclusions: We showed that collecting cost data alongside field activities is both feasible and immediately informed by implementation of interventions on the ground. The strategy offers unique opportunities for collecting granular data to support a range of policy questions toward optimizing health interventions and facilitating transfer of economic evidence from the field to the program.

For peer review only - http://bmjopen.bmj.com/site/about/guidelines.xhtml

For peer review only

Article Summary

Strengths and limitations of this study

- We demonstrate that it is both feasible and advantageous to collect cost data alongside trial field activities.
- The costing strategy detailed here ensured high recall, supported collaboration between trial teams, and grounded costing in operational insight.
- The approach facilitated tailoring of data collection to different countries without undermining consistency of estimates between sites.
- While the strategy yielded high-resolution data, it was labor intensive and required technical expertise to derive costs from the data collated.

For peer review only

Introduction

Field trials conducted outside of clinical settings generate important evidence for making public health decisions and optimizing health programs. They indicate what the impact of efficacious interventions might be under real-world conditions, within representative target populations, and with implementation processes that aim to inform routine programmatic activities[1]. Increasingly, economic outcomes, including costs and cost-effectiveness, are evaluated within community-based research studies to generate evidence regarding sustainability and scalability of tested interventions[2-6].

While methodological guidance on economic evaluation and valuation of costs has been clearly outlined[7-10], guidance on practical aspects of cost data collection in the field is relatively scarce[11]. Normative documents, including the recently released Reference Case for Global Health Costing[10], cover some aspects of cost data collection including the perspective to take, the level at which to conduct costing, and offer guidance on cost classification categories to adopt for presenting results of the study. However, apart from a brief overview of approaches for planning a costing exercise and potential data sources, these guidelines are limited in their description of strategies for integrating costing studies within field trials. A number of technical documents, targeted primarily at program managers, offer guidance on budgeting of health interventions[9, 12-15]; these program-specific guidelines emphasize retrospective evaluation of interventions directed by costing templates or interactive tools. Guidelines for cost data collection along clinical trials, on the other hand, support ongoing costing activities[16, 17]. Although primarily aimed at developed country contexts, these documents discuss a number of considerations relevant for field evaluations such as the need to isolate trial-driven resource use, the importance of prioritizing data collection toward resource line items that are most likely to change the policy decision (i.e. if costing is to support cost-effectiveness analyses), and integration of costing within the routine trial reporting. Finally, guidelines on costing health interventions in low- and middle-income countries address specific challenges that arise due to poor data availability and limited economic expertise in these countries[18, 19]. The guidelines further highlight the trade-offs between accuracy and data availability and suggest use of mixed methodologies (i.e. gross costing along with bottom-up approaches) to tackle these limitations.

Integrating costing within a multi-country field trial raises basic practical questions not adequately addressed by this literature. How to assign roles for cost data collection and analysis, when to collect the data and for how long, what level of detail is appropriate and can be feasibly accommodated in the context of a field study, which tools to use, how to balance specificity of sites with the need for consistent comparison of costs between sites,

For peer review only - http://bmjopen.bmj.com/site/about/guidelines.xhtml

how best to accommodate multiple economic endpoints and so on. Choices made by researchers to address these questions have important implications for the cost estimates derived and for their relevance for policy decisions the trial ultimately aims to inform. Somewhat surprisingly, there has been remarkably little exchange between practitioners on their experience with different strategies for costing interventions in the field[19, 20].

In this paper, we present an approach for incorporating cost data collection within the DeWorm3 project – a community-based cluster randomized trial testing the feasibility of interrupting transmission of soil-transmitted helminth (STH) infections in India, Benin and Malawi[2, 21]. This project is funded by the Bill & Melinda Gates Foundation through a grant awarded to the Natural History Museum (NHM). The DeWorm3 interventions consist of bi-annual community-wide mass drug administration (cMDA) targeting eligible individuals of all ages compared to standard of care deworming of school-age children (SAC). The trial is conducted in focal geographic areas with a population between 100,000 to 140,000 residing in each country site. CMDA is deployed for three years (six rounds) followed by two years of monitoring for recrudescence. The project aims to generate evidence for local and global stakeholders on optimal strategies for control and elimination of STH, including multi-level factors influencing treatment coverage[21].

Although the trial and its evaluation, including costing, are ongoing, we share our early experience with the methodology in order to stimulate an exchange among practitioners on the design and implementation of costing studies in the field. We discuss here some of the practical choices made and ways in which these decisions were motivated by the aims of the project, the infrastructure, and the implementation of the trial interventions in specific sites. We report preliminary cost estimates for the India trial site and reflect on the challenges encountered in implementing our costing strategy, the ways in which we addressed these, and highlight the lessons learned for future costing studies operating in similar contexts.

Methods

Interventions

The current global strategy for STH control relies on MDA in SAC[22]. The frequency and targeting of MDA campaigns are determined by the prevalence of infection in SAC with more frequent treatment where prevalence is high[23]. The Indian STH program distributes MDA with Albendazole to pre-SAC and SAC children via a bi-annual national mass campaign called the National Deworming Day (NDD)[24]. Drugs are administered during school days by teachers under the supervision of the local medical staff and the Ministry of Health (MoH).

For peer review only - http://bmjopen.bmj.com/site/about/guidelines.xhtml

cMDA programs for STH control, on the other hand, target eligible community members of all ages. In India cMDA in DeWorm3 intervention clusters started a week after NDD; drugs were administered to all individuals who were not treated in schools (i.e. adults and out-of-school children). Prior to drug distribution, the DeWorm3 staff sensitized the community by distributing IEC materials, designed based on the NDD campaign with additional artwork depicting the treatment of whole communities, and by making public announcements. Community Drug Distributors (CDDs) were recruited from community volunteers and Accredited Social Health Activist (ASHA) workers. CDDs and project field officers supporting drug distribution were trained by the DeWorm3 team on consent seeking, drug administration, and data collection. A medical team was trained on adverse event monitoring. During the campaign rounds, CDDs were accompanied by project field officers and supervised by project staff. CDDs were paid a daily per-diem and received a mobile data package for each campaign round. Additionally, health facilities received an annual honorarium payment for health center staff that supported the project on site. MDA coverage was assessed in both intervention and control clusters by field officers using mobile devices equipped with SurveyCTO[25]. Data were uploaded to a central database and analyzed by the trial data management team. Mop-up drug distributions were conducted in all clusters in all rounds. A detailed description of operational activities and resource use supporting cMDA is presented in SI Tables S1-2.

Study purpose, perspective and scope

The purpose of the costing study was to determine the incremental resource requirements necessary for expanding MDA for STH to target individuals of all ages and to feed into the cost-effectiveness analysis of the DeWorm3 interventions[21]. To align with these aims, the societal perspective was taken for the evaluation, and an assessment of both financial (most relevant for NTD programs and short-term planning) and economic costs (most relevant for MOH and global stakeholders) was undertaken (See SI Box S1 for key terms). Costs were evaluated around an operational intervention model following the micro-costing approach[26]. This methodology, while effort-intensive, grounds the evaluation in the implementation process and yields high resolution data to inform modeling of cMDA modalities and contexts beyond those observed in the trial.

Integration of costing within the DeWorm3 project

Integrating costing within a clinical trial required deciding who will collect the data, when, and for how long. The DeWorm3 project included local institutions with strong epidemiological and clinical field research expertise (Table 1). Capitalizing on the interest of these organizations to develop capacity for economic evaluation, in-country teams were designated

For peer review only - http://bmjopen.bmj.com/site/about/guidelines.xhtml

to lead cost data collection using standardized tools and with support from off-site health economics research groups. Ensuring country ownership was central to DeWorm3 design, as the project aims to facilitate transfer of knowledge from the field to support STH programs and policies in countries hosting the trial.

Table 1. Implementation of DeWorm3 trial at study sites

Location	Grant-holding organization	Implementing organization and partners	National Standard of Care STH Control Strategy (age target)	Community-wide STH MDA	Primary costing point person(s)
Come Commune, Benin	Institut de Recherche Clinique pour Développement	- Institut de Recherche Clinique du Benin<br>- Institut de Recherche Clinique pour Développement<br>- Ministry of Health, Benin	School-based MDA (5-14 years old)	Bi-annual community-wide MDA in all ages	Accountant, Assistant accountant, IS lead
Tamil Nadu State, India	Christian Medical College, Vellore	- Christian Medical College, Vellore<br>- Ministry of Health and Family Welfare, India	School-based MDA and National Deworming days (1-19 years old)	Bi-annual community-wide MDA in all ages; as a mop-up following NDD	Grant manager, Trial coordinator
Mangochi District, Malawi	Blantyre Institute for Community Outreach	- Blantyre Institute for Community Outreach<br>- London School of Tropical Medicine and Hygiene<br>- Ministry of Health and Education, Malawi	School-based MDA and National Deworming days (1-14 years old)	Bi-annual community-wide MDA in all ages	Accountant, Field accountant, Field manager

DeWorm3 is a hybrid trial, which incorporates implementation and operational questions into a clinical trial design[27]. Since costs are an implementation outcome, the costing and economic analyses were considered under the umbrella of implementation science (IS) research (Figure1). The IS studies collected data on operational processes, health systems and policy contexts, and community experience with cMDA. Thus the data collection for the costing study could narrowly focus on direct financial and economic costs of cMDA to the project.

<Figure 1>

For peer review only - http://bmjopen.bmj.com/site/about/guidelines.xhtml

The costing study covered all field activities (Figure1, black and blue panels). This removed the need for cost data collection point persons at each site to decide which expenditures to record and allowed for a comprehensive assessment of resources that contributed to cMDA coverage and, ultimately, intervention effectiveness. Moreover, by tracking resources beyond operational activities immediately supporting cMDA, the study collated inputs for modeling costs of programmatic components such as surveillance and monitoring that are part of routine NTD control (i.e. population census and prevalence surveys).

Cost data were collected over two years (four MDA rounds) in order to capture improvements in efficiency of the program as teams gained operational experience deploying the intervention over multiple rounds.

Integration of costing within the trial sites

The key feature of our study design is the delineation of responsibilities for data collection and evaluation between the collaborators. Costing teams at each site were responsible for identifying resources used to support implementation of trial activities, extracting information on their respective prices and quantities, and recording these data using standardized data collection instruments. The off-site economics team, on the other hand, was responsible for triangulating these data to value resources in economic terms.

We conducted several rounds of training and formative meetings with site-designated costing point persons and other supporting staff. Several instruments, described briefly in Table 2, were developed to guide cost data collection. These tools included an Excel-based Costing Tool, an Activity Table, and an Activity Calendar. The Costing Tool detailed by activity quantities, prices, and costs of the resources used, while the Activity Table described implementation of activities costed in the field covering additional resources for which no expenditures were incurred. Data entry templates from the Costing Tool and an example of a filled out Activity Table for India site are shared in the Supplementary Information file (SI File S1 and Table S2).

Table 2 DeWorm3 instruments for collecting inputs to economic analysis

Costing instrument	Type of cost	Content	Responsible Person	Location of responsibl e person
Activity Table*	Financial and Opportunity	Description of operational activities and sub-activities, number of project staff and other resources used, number of days	Grant Manager, Trial coordinator	Field

For peer review only - http://bmjopen.bmj.com/site/about/guidelines.xhtml

Activity Calendar		Start, end dates, and duration of operational activities	Field manager, Trial coordinator	Field
Costing Tool	Financial	Resource line items, corresponding prices, quantities, and expenditure recorded by sub-activity; separate modules for start-up and implementation	Grant manager, Trial coordinator	Site
Spend Report	Financial	Trial expenditures	Project financial manager	External
Central Expenditure Report	Financial	List of prices and quantities of items procured at the central level	Project financial manager	External

*An example of an Activity Table detailing a subset of trial activities implemented related to cMDA is enclosed in SI, Table S2

In the Costing Tool, resources were tracked differently by stage of implementation, specifically distinguishing between planning and other pre-trial activities (i.e. start-up) and activities taking place during the trial itself. For the start-up phase, sites reported the number of meetings, time dedicated to planning, sensitization of country stakeholders, and development of training and sensitization materials for the trial. In the implementation stage, in addition to resource lists and quantities, information was collected also on prices, expenditure and other details relevant to economic valuation (e.g. year of purchase for capital items). The implementation modules of the Costing Tool further distinguished between resources that contributed to multiple trial activities (shared resources) such as trial PI, facilities, vehicles, etc. from resources that contributed to a single activity. Shared resources were recorded in the Costing Tool at the start of the trial and were reviewed for completeness at each round of data collection. Thus for each trial activity, only the incremental expenditures (i.e. those additional to program overheads and capital) were recorded by sites, thus minimizing the effort and facilitating allocation of expenditure data.

Institutional differences and differences in technical capacity between sites required tailoring of the cost data collection strategy to each partner. Where reporting was centralized, one costing point person was designated with cost data collection, where different staff were responsible for dispensing funds for specific operational activities or resources (i.e. fuel or bulk purchases of supplies) multiple staff assumed responsibility for compiling cost data at their respective levels. During piloting visits, the economics team, working together with in-country partners, mapped the costing strategy into sites' routine financial reporting, tailored to the site the costing instruments developed, and updated standard operating procedures that outlined roles, responsibilities and reporting timelines for costing at the site.

For peer review only - http://bmjopen.bmj.com/site/about/guidelines.xhtml

As an illustrative example, Figure2 shows the flow of funds and financial reporting at DeWorm3 India site. The site designated their grants manager to lead cost data collection. Guided by the Costing Tool, the grants manager extracted information from receipts originating from the project's central offices, the Christian Medical College (CMC), Vellore which hosted the trial, and the field sites. Expenditures that were incurred outside of the trial site (i.e. bulk purchases of commodities and supplies by NHM DeWorm3 central team) were sourced and added to the Costing Tool in the analysis stage. Integrated within the trial operations, the costing study benefitted from sites' established financial reporting processes, quality control, and oversight by partner institutions.

<Figure 2>

From costing inputs to an estimate of intervention costs

Separating cost data collection from valuation of resources meant that the bulk of data cleaning, categorization, consistency checks, and validation happened during data quality and assurance checks at the central level. Entries in the Costing Tool were first evaluated for completeness and then for consistency by comparing expenditures and resource use against activities implemented as detailed in the Activity Table. During data quality checks, we standardized descriptions of resource line items, cost categories, and activities to allow comparisons within and between different activities at each site. We checked for duplicate entries, ensured that the same resources were priced at the same rate, and that similar activities yielded comparable costs. Where inconsistencies were identified, site teams provided additional operational details and clarifications to resolve the issue, and brainstormed on whether adjustments to the analysis were needed to reflect the implementation.

Once cleaned, we applied standardized programming routines, implemented in Stata[28], to convert these data into estimates of cMDA and trial costs. Specifically:

1. **Start-up costs:** We retrospectively estimated costs of planning and other pre-trial activities for which no expenditure data were recorded. In the Costing Tool these were described according to the type (i.e. sensitization meetings, micro-planning) and intensity of activities conducted (i.e. the number of meetings). We used information from other sections of the Costing Tool (i.e. project staff wages, transportation) to value the respective resource use via direct allocation. Start-up costs were treated as capital items and were annualized over the fiver year duration of the trial.

For peer review only - http://bmjopen.bmj.com/site/about/guidelines.xhtml

2. **Activity costs:** Costs of trial operational activities were obtained by summing costs of the resource line items recorded in the Costing Tool and adding on shared resources and program overheads. Resource line items were valued according to their respective cost category (SI Table S3) applying, where appropriate, annualization and adjusting for percentage used for the trial to estimate the value of these resources for the first cMDA cycle.

3. **Shared costs:** Shared resources that could be allocated to specific activities and sub-activities such as laboratory equipment were allocated accordingly, while the rest (primarily research and management staff and overheads) were grouped under "Program management" cost category (SI Table S4). Resources that contributed to multiple activities, such as some transportation-related expenses were allocated based on the number of activity days.

To derive cost of cMDA, we isolated cMDA-related expenditures from all other trial expenditures. For the most part, the distinction between intervention and research costs was tied to activity or sub-activity category (i.e. expenditures related to training of CDDs supporting MDA were categorized as cMDA-related). If, however, an activity or a resource category contributed to both cMDA and research activities, in some sites, was the case for training of field workers, costs were allocated based on the ratio of cMDA days to total trial days. For some shared resources, costs were allocated multiple times: first to the respective activity and then to cMDA or the trial (SI Table S4). Total cost of cMDA was then obtained by summing over sub-activities supporting drug administration, and adding on cMDA-attributable share of planning and program costs.

Differences in implementation of the costing approach between sites required further tailoring of these standardized valuation and allocation routines. In India, for instance, compensation of field staff and some other field expenditures (i.e. materials and supplies, some fuel and transportation costs) were tracked on a monthly, rather than weekly or daily, basis. These monthly expenditure totals often covered multiple activities each requiring a varying number of field staff. To allocate these, we first calculated a daily equivalent value for each expenditure type and, where possible, staff category. Specifically, monthly expenditures were summed over the 12-month cycle within which the two rounds of cMDA were delivered and divided by the total number of staff compensated each month times 20.5 (average number of working days per month according to India country team). We then mapped these monthly expenditure categories into trial activities based on Activity Table, flagging activities to which

For peer review only - http://bmjopen.bmj.com/site/about/guidelines.xhtml

a specific staff or resource category contributed. Finally, activity totals were obtained by multiplying the daily equivalent value for each expenditure type by the number staff and the number of days over which the respective activity was conducted. To the extent that multiple activities, including those research-related, were conducted simultaneously by the field staff this strategy might have overstated total cMDA-attributable spending.

Costs of cMDA in the DeWorm3 India site

To support a critical assessment of the study design presented, we share preliminary costs for the first year of cMDA in the DeWorm3 India site. These are interim findings that only integrate financial expenditures at this time, and final cost estimates from DeWorm3 are forthcoming. The average financial cost of cMDA were summarized by key operational activities, including start-up and program overheads. Finally, we considered how our results from the trial might translate to costs under routine programmatic implementation by Indian NDD program.

Results

Table 3 presents costs for two rounds of cMDA and cMDA-supportive activities conducted at the India DeWorm3 site between October 2017 and September 2018. In the first year these averaged about $1.14 per person treated per round. While the unit costs were virtually identical between the two rounds, there were differences in the number of field staff (lower in second round) and the number of implementation days for sensitization, training, and drug distribution campaign (higher in the second round). Drug administration in the community accounted for over a quarter of total intervention cost (row "cMDA", Table 3). As drugs were donated, cMDA costs were primarily driven by personnel: wages of field staff and incentives to CDDs accounted of about 70% of total costs (SI Table S5). The second largest cost component was "Program management and overheads" (33% of total cost). This cost category covers wages of core trial personnel and expenses related to transportation for supportive supervision or other activities conducted by these management cadre. Program overheads also include data processing and data management costs (i.e. related to registration of households for MDA).

For peer review only - http://bmjopen.bmj.com/site/about/guidelines.xhtml

Table 3 First year financial costs of STH cMDA* implementation in India DeWorm3 trial site, USD 2018

	Total (US$)			Average cost Profile (%)	Average cost per person treated per round (US$)	Average cost per Capita per round (US$)
MDA round	1	2	1+2	1+2	1+2	1+2
TOTAL	60,320	54,305	114,626	100.0	1.14	0.84
*TOTAL incremental***	*4,829*	*6,577*	*11,406*	*10.0*	*0.11*	*0.08*
Start-up activities***	1,094	1,055	2,149	1.9	0.02	0.02
Program management	21,300	20,540	41,840	36.5	0.42	0.31
Drug testing and distribution to site	304	331	636	0.6	0.01	0.00
CDD recruitment	1,066	1,028	2,095	1.8	0.02	0.02
Community sensitization	12,924	9,343	22,267	19.4	0.22	0.16
CDD bags and job aids	555	428	983	0.9	0.01	0.01
Banners, posters	969	969	1,937	1.7	0.02	0.01
Training	5,003	2,840	7,843	6.8	0.08	0.06
Travel allowance for CDDs	240	170	409	0.4	0.00	0.00
Refreshments for CDDs	10	17	26	0.0	0.00	0.00
cMDA	13,442	14,659	28,101	24.5	0.28	0.21
CDD incentives	2,632	4,577	7,210	6.3	0.07	0.05
CDD mobile allowance	120	85	205	0.2	0.00	0.00
Coverage survey	3,116	2,451	5,567	4.9	0.06	0.04
Mop-up	2,070	2,058	4,128	3.6	0.04	0.03

*cMDA was implemented as a mop-up following NDD campaign that targeted school-aged children; **Incremental costs represent a subset of rows highlighted in *cursive*, see text for details. *** Start-up activities annualized over the duration of the trial (5 years). Grey shaded rows are a subset of the higher level activity grouping. Total number of people treated in first round of cMDA (cMDA1) was 51’320 (site total population 68’442); in second round (cMDA2) – 49’488 (site total population 68’460); total treated over the two rounds (cMDA1+cMDA2) was 100'808 (total population 136'902). Costs were converted to USD using average annual exchange rate over the study period (1 INR=0.01462 USD) [29]. STH= Soil-Transmitted Helminth infections; cMDA= community-wide Mass Drug Administration; CDD= Community Drug Distributor.

For peer review only - http://bmjopen.bmj.com/site/about/guidelines.xhtml

Due to the large contribution of field staff to the overall cost of cMDA, the estimates were highly sensitive to decisions on how these resources were quantified and valued. The estimates presented in Table 3 above were obtained by applying a fixed monthly base rate to the number of staff and activity days to derive total cost. In practice, though, there was some variation around the actual rate paid out to field staff. At the India site, field workers were borrowed from other studies to assist in drug distribution. These additional staff were compensated at their regular rate which was below the rate paid in the DeWorm3 project. Moreover, during the campaign, field staff also worked on weekends and through holidays earning overtime. To better capture the value of field staff we re-ran the analysis applying an adjustment weight to the base wage. The site staff-level weight was calculated as a ratio of the respective monthly base rate to monthly equivalent rate derived from site expenditure data. It varied from 0.9 to 2.5 between staff categories: nearly identical to the monthly base rate for field officers (weights ranging between 0.9 and 1.01) and substantially above the base rate for field managers and supervisors indicating overtime. One weight was then applied to all sub-activities that started in a given month thus inflating the base wages depending on expenditures incurred in a given month. After this rate adjustment the average cost per person treated per round increased to $1.20 (SI Table S6).

Arguably, from the perspective of the program the primary interest is in the incremental resource needs for reaching adults and children not treated under the current NDD deworming campaign. Thus the relevance of the totals reported for the trial above and some of the resource line items costed warrant further consideration. For example, it could be suggested that planning, program management, and community sensitization activities would not necessarily change to accommodate the broader age target for cMDA. Provided there is spare capacity in the system, costs related to drugs, drug storage and distribution, and also coverage surveys and mop-up, would scale linearly with the number of people covered. The incremental costs, compared to the current SAC-targeted strategy, would then be incurred for recruitment, training, and incentives of/to CDD's (See Table 3 rows highlighted in cursive). This scenario adds up to a significantly lower cost estimate per person treated of $0.11, compared to $1.14 reported from the perspective of the trial. Further analyses with input from the program, however, are needed to appropriately contextualize these findings.

Recognizing that comparisons of costs between studies are often limited due to differences in the objectives of respective evaluations, scope of resources costed as well as methodological choices made by analysts[10], these comparisons, nonetheless, provide additional support to the methodology described here. We found that the cost estimates derived align reasonably well with cMDA costs reported elsewhere. For instance, a recent

For peer review only - http://bmjopen.bmj.com/site/about/guidelines.xhtml

multi-site study from African region reported an average of $1.75 per person treated per round of community-wide MDA treatment[30]. A lower estimate of $0.65[1] per person treated was reported over a 2 year program in 4 districts in Niger, this is about half the estimate of drug delivery for SAC in the same study[31]. The scale of the program: 300,000 to over 450,000 people treated in Niger compared to just under 70,000 in the DeWorm3 India site explains some of the difference in costs between the two studies. Another estimate comes from a meta-regression of MDA costs for NTDs; net of community volunteers and drugs, the estimate averaged $0.40 (range $0.02 to $2.90) per person per round[32], which is comparable to $0.58 in this study when we exclude payments to CDDs and field officers.

Discussion

The costing strategy presented here was developed in close collaboration with trialists both at and off-site that helped us adapt the strategy to the technical and operational realities at sites. The methodology capitalized on the immediacy of resource use on the ground, the operational expertise and established processes for routine financial reporting within the implementing organizations. Integrating costing within the broader scope of the trial, on the other hand, allowed us to build on other research studies conducted concomitantly to get a fuller appreciation of resource use related to cMDA implementation. We share below lessons learned designing and implementing the methodology in the DeWorm3 trial.

Balancing flexibility and consistency in designing the strategy

One of the key challenges in conducting a multi-country cost analysis is in balancing the need for flexibility to accommodate the trial and intervention features in a given setting with the need for consistency and standardization of cost estimates across the trial sites. Our reliance on the micro-costing methodology and use of multiple instruments to detail operational activities on the ground, including resource inputs, helped capture these differences. Piloting of the tools, and their tailoring to and integration into routine financial reporting further ensured that robust estimates could be derived despite differences in sites contextual features. Adapting data collection to sites, however, did complicate the analysis. We could ensure consistent valuation of resources by relying on programmed routines that fixed *how* resources were categorized, priced, annualized, and allocated to the intervention across sites.

[1] Niger GDP deflator applied to 2005 estimate of $0.46 reported in the study[10]

For peer review only - http://bmjopen.bmj.com/site/about/guidelines.xhtml

Harnessing synergies and fostering collaborations within project

The complexity of the DeWorm3 project posed a number of its own challenges for the design and implementation of the costing study. With many research activities conducted alongside the intervention (e.g. annual census, prevalence surveys), developing a costing strategy required coordination between the many project partners on and off-site. From decisions on who will lead the cost data collection to when to pilot the costing tools, to how best to monitor quality of the data collected - all had to be agreed upon between multiple parties. The communication overhead was minimized through integration of costing under the broader IS research agenda; additionally, reporting was further streamlined by centralized tracking of costing outputs by the IS team which formally tied costing deliverables to funding transfers to sites.

While conceptually, we could identify synergies between studies in the design stages of the project, differences in timing of data collection and processing meant that we could not fully avoid some duplication of effort. For instance, Activity Table compiled by field staff (i.e. field manager or trial coordinator) at each site collated some of the same information as IS process mapping exercises. Activity Table filled out by the field staff during each trial activity was immediately available to the costing point person at the site and the economic teams at submission of costing instruments; the IS outputs, populated with data from multiple study clusters, required time for data collection and processing before these could be integrated. On the other hand, the overlaps between data collection instruments allowed for triangulation of data in the analysis stage, which further strengthened the quality of the evidence.

Success of the costing strategy further relied on close cooperation between the different study leads at the site. Efficient communication and management within the implementing agency meant that it was possible for the designated field staff and costing point persons to validate resource allocation to specific activities and reflect those appropriately in the costing instruments. When sites were limited for time in collecting or reviewing costs, higher levels of aggregation of expenditure data were recorded.

Challenges and opportunities in country-led cost data collection

We found that we could support site data collection efforts best when our guidance was grounded in an understanding of operational details of interventions being evaluated and the specific contexts in which the evaluation was conducted. To this end, delaying site visits and piloting of tools until field activities have commenced appeared to have been one of the critical choices we made in implementing the strategy. This ensured that both financing

For peer review only - http://bmjopen.bmj.com/site/about/guidelines.xhtml

channels and routine reporting at sites were established by the time of the visit and we could map these together with costing leads to guide where and how best to collect the data.

Our initial guidance on selecting the focal person for cost data collection was an on-site team member that was primarily field-based and had a part-time duty to populate cost data collection instruments. However, due to the sensitivities of financial reporting, particularly of salaries, it was challenging to designate anyone other than accounting staff to collate economic data. This naturally imposed a substantial burden on accountants already fully engaged in their primary duties. To make it possible for an accountant, removed from the field, to lead cost data collection it was necessary to develop additional instruments (i.e. Activity Table) and to facilitate a closer involvement of field staff with the costing study (i.e. via Activity Table, Activity Calendar) than originally anticipated. On the other hand these additional tools enabled unique insights into implementation of interventions on the ground and facilitated quality control and subsequent analyses of the data collected.

At the same time routine financial reporting at sites offered some unanticipated opportunities for cost data collection. For example, in Malawi, weekly financial advances were made by an off-site accountant to the field team which were reconciled the following week against a filled-out Expenditure Ledger. The latter, organized in an Excel table, itemized field expenses detailing prices, quantities along with a brief description for each entry. Both the frequency of recording and the level of detail at which expenditures were described in the Ledger made it easy to integrate this site-developed tool into the costing strategy.

Some of the project research studies were conducted either concomitantly with or relying on resources that also supported cMDA. Allocation of shared resources is an area that requires value judgements which are often difficult for those new to costing. There are several aspects of our strategy that made these choices less arbitrary. First, extending the scope of the evaluation to all trial activities (including research-related) removed the need for local teams to decide which activities or resources to track. Second, capital resource line items and other resources that contributed to multiple activities like program staff were explicitly identified and tracked separately in the Costing Tool, thus focusing data collection on tracking incremental expenditures for each activity and leaving allocation of shared resources to the economics team.

Separating data collection and valuation of resources ensured that any inconsistencies in data entry could be resolved in the analysis stage. This feature proved to be particularly advantageous as, despite multiple points of validation at the field site, entries in the Costing

For peer review only - http://bmjopen.bmj.com/site/about/guidelines.xhtml

Tool were often misallocated between activities. These challenges highlight limitations of pre-populated costing tools that collate aggregated expenditures recorded by field teams into an estimate of unit costs. We were able to resolve these errors by triangulating information across the costing instruments and by comparing entries between the trial sites; being able to follow-up with teams shortly after an activity was completed to validate any corrections to the data ensured high recall.

Sites also struggled with the level of detail for cost data collection. Modifying internal reporting templates to include information on prices, quantities, and activities to which an expenditure/ resource line item contributed made it easier for sites to capture these data. However, it required more time to fill-out these documents. Where there were multiple financially responsible persons the volume of receipts, particularly those attesting to petit-cash spending or other low-level expenditures, created a substantial hurdle in recording and transferring these data into costing tools.

Sites voiced difficulties mapping expenditures in the Costing Tool into their internal accounting documents. To allow sites to double check expenditure totals and to ensure that expenditures were accurately and comprehensively reported in both systems we included an additional column in the Tool for sites' internal transfer number. These linked resource line items in the Tool to the internal financial tranche that paid for it, which further improved reporting.

We found that our approach worked best in sites where there was an established and lean process for financial reporting within the implementing organization. Where bureaucracy was heavy, for instance, when reporting was required for multiple funders each with own accounting system there was low capacity to support data collection for the costing study. Multiple staff were required to facilitate the process and to transfer financial and resource use data into a format that could be used for the economic analysis.

Relevance to programs

The cost estimates of cMDA implementation presented here are only the first step in the economic analysis. Most immediately, the scope of the evaluation will expand to consider the full economic costs of the program and broaden the perspective to include indirect costs to the community and the project by triangulating data from surveys conducted along the trial (Figure1). Informed with IS research, costing will further explore cost drivers and heterogeneity in cost of cMDA within and between sites. In India, the analysis will compare costs between two study sub-sites that differ in their population, socio-cultural and economic

For peer review only - http://bmjopen.bmj.com/site/about/guidelines.xhtml

characteristics, and geography. IS research will further help identify the incremental resource use for current and alternative cMDA implementation scenarios to feed back to programs.

The operational model for cMDA implementation in the trial detailed in Activity Table forms the basis for an assessment of the delivery modality within the project to what delivery might look like when scaled-up by the program. Using the data collected, we can begin to isolate aspects of intervention design that are trial-driven and update these with assumptions reflecting operational realities of the program. When presenting cost estimates for the trial we hypothesized the potential scope of incremental resources that might be relevant to India NTD program, the resulting estimate, describing primarily incentives to CDDs and community sensitization aids, was drastically different than the one describing the full scope of resources that supported cMDA implementation in the trial (i.e. $0.11 compared to $1.14 per person treated per round). The methodology thus facilitates careful interpretation of the costs derived and supports effective communication of findings to the national program recognizing the many ways in which the scalable model might differ from the one piloted and the likely cost implications of these differences[17, 33].

Conclusion

Field research produces important insights about effectiveness of interventions in the community. The programmatic relevance of field trials can be further strengthened by integrating costing within the trial study design. We showed here that collecting cost data along field activities is both feasible and immediately informed by implementation of interventions on the ground. The strategy presented offers unique opportunities for collecting high resolution data to support a broad range of policy questions toward optimizing health interventions and facilitating transfer of economic evidence from the field to the program.

For peer review only

Figure legends

Figure 1. Integration of costing study within the DeWorm3 trial research activities.

The figure illustrates key surveys (top blue panel) and IS studies (bottom green panel) conducted along cMDA in the DeWorm3 trial. It maps cost-effectiveness (to which costing contributes) within the IS research and highlights the contribution of other studies to costing and broader economic research agenda of the project. For further details refer to SI Table S1. Figure adapted from presentation by Means et al "DeWorm3 Project 2017 Annual Site PI Meeting." 2017.

Figure 2. Routine financial reporting at India DeWorm3 trial site

The figure illustrates the flow of funds and financial reporting at India DeWorm3 trial site. Project's grants manager – center green box – is the focal point through which all country funds supporting the trial flow. For each project level the diagram shows staff responsible for dispersing funds (left) and gives examples of some of the resource line items that are purchased/ consumed/ recorded at that level (right panels). Bottom row – field site – additionally highlights itemized tracking of expenditures related to field worker per-diems and fuel (important cost driver of MDA activities).

Conflict of interest

The authors declare no conflict of interest.

Author contribution

KG, ARM, FT designed data collection tools; KG piloted data collection tools; SPG, SPK collected data and contributed to design of data collection tools; CM, MS conducted data queries; KG developed data analysis and validation routines; KG, CM analyzed the data; KG wrote the first draft; KG, ARM, CM wrote the manuscript. MS, FT provided critical inputs and edits to the manuscript. KG, MS, SPG, SPK, CM, SSRA, JW, ARM, FT reviewed and approved the final version of the manuscript for publication.

Funding

This project was funded by a sub-contract from the Natural History Museum grant from the Bill and Melinda Gates foundation OPP1129535.

Acknowledgements

We would like to acknowledge in-country teams and off-site project partners who generously contributed their experience and insight to the design of the costing study and who continue to support cost data collection within the DeWorm3 trial. Specifically, Wongani Lungu, Chikondi Chikotichalera, Roselyn Hara, Euripide Avokpaho, Nadege Aicheou, Monrenike Bada, James Simwanza, David Chinyanya, Rajesh Kumar Rajendiran, Chinnaduraipandi Paulsamy, Elodie Yard, Stefan Witek-McManus, Katherine E Halliday, Hugo Legge, Leanne Doran, William E Oswald, Fabian Schär, Marie-Claire Gwayi-Chore, Mira Emmanuel-Fabua, Iain Gardiner, Moudachirou Ibikounle, Khumbo Kalua, Adrian JF Luty, and Robin Bailey.

For peer review only - http://bmjopen.bmj.com/site/about/guidelines.xhtml

References

1. In *Field Trials of Health Interventions: A Toolbox.* Edited by rd, Smith PG, Morrow RH, Ross DA. Oxford (UK)2015: *Wellcome Trust-Funded Monographs and Book Chapters*].
2. Asbjornsdottir KH, Ajjampur SSR, Anderson RM, Bailey R, Gardiner I, Halliday KE, Ibikounle M, Kalua K, Kang G, Littlewood DTJ, et al: **Assessing the feasibility of interrupting the transmission of soil-transmitted helminths through mass drug administration: The DeWorm3 cluster randomized trial protocol.** *PLoS Negl Trop Dis* 2018, **12:**e0006166.
3. Pullan RL, Halliday KE, Oswald WE, McHaro C, Beaumont E, Kepha S, Witek-McManus S, Gichuki PM, Allen E, Drake T, et al: **Effects, equity, and cost of school-based and community-wide treatment strategies for soil-transmitted helminths in Kenya: a cluster-randomised controlled trial.** *Lancet* 2019, **393:**2039-2050.
4. Pitt C, Ndiaye M, Conteh L, Sy O, Hadj Ba E, Cisse B, Gomis JF, Gaye O, Ndiaye JL, Milligan PJ: **Large-scale delivery of seasonal malaria chemoprevention to children under 10 in Senegal: an economic analysis.** *Health Policy Plan* 2017, **32:**1256-1266.
5. Means AR, Kemp CG, Gwayi-Chore MC, Gimbel S, Soi C, Sherr K, Wagenaar BH, Wasserheit JN, Weiner BJ: **Evaluating and optimizing the consolidated framework for implementation research (CFIR) for use in low- and middle-income countries: a systematic review.** *Implementation Science* 2020, **15**.
6. Proctor E, Silmere H, Raghavan R, Hovmand P, Aarons G, Bunger A, Griffey R, Hensley M: **Outcomes for implementation research: conceptual distinctions, measurement challenges, and research agenda.** *Adm Policy Ment Health* 2011, **38:**65-76.
7. Walker D: **Cost and cost-effectiveness guidelines: which ones to use?** *Health Policy and Planning* 2001, **16:**113-121.
8. Drummond MF, Sculpher MJ, Torrance GW, O`Brien BJ, Stoddart GL: *Methods for the economic evaluation of health care programmes.* 3 edn. Oxford, United Kingdom: Oxford University Press; 2005.
9. Phillips MA, Mills A, Dye C: **Guidelines for cost-effectiveness analysis of vector control.** In *PEEM Guidelines Series 3*. Geneva, Switzerland1993.
10. Vassall A, Sweeney S, Kahn J, Gomez GB, Bollinger L, Marseille E, Herzel B, DeCormier Plosky W, Cunnama L, Sinanovic E, et al: **Reference Case for Global Health Costing.** 2016.
11. Crowell V, Levin A, Galactionova K, Tediosi F: **Costing and budgeting for malaria service delivery.** Basel, Switzerland: Swiss Tropical and Public Health Institute; 2013.
12. Creese A, Parker D: **Cost analysis in primary health care: a training manual for program managers.** Geneva, Switzerland1994.
13. UNAIDS: **Workbook for collection of Cost Information of HIV Services.** 2011.
14. UNAIDS: **Costing Guidelines for HIV Prevention Strategies.** Geneva: Joint United Nations Programme on HIV/AIDS; 2000.
15. Immunization VaB: **WHO-UNICEF guidelines for developing comprehensive multi-year plan (cMYP).** 2014.
16. Doshi JA, Glick HA, Polsky D: **Analyses of cost data in economic evaluations conducted alongside randomized controlled trials.** *Value in Health* 2006, **9:**334-340.
17. O'Sullivan AK, Thompson D, Drummond MF: **Collection of health-economic data alongside clinical trials: Is there a future for piggyback evaluations?** *Value in Health* 2005, **8:**67-79.
18. Hendriks ME, Kundu P, Boers AC, Bolarinwa OA, te Pas MJ, Akande TM, Agbede K, Gomez GB, Redekop WK, Schultsz C, Tan SS: **Step-by-step guideline for disease-

For peer review only - http://bmjopen.bmj.com/site/about/guidelines.xhtml

specific costing studies in low-and middle-income countries: a mixed methodology. *Global Health Action* 2014, **7**.
19. Batura N, Pulkki-Brannstrom AM, Agrawal P, Bagra A, Haghparast-Bidgoli H, Bozzani F, Colbourn T, Greco G, Hossain T, Sinha R, et al: **Collecting and analysing cost data for complex public health trials: reflections on practice.** *Global Health Action* 2014, **7**.
20. **The cMYP Immunization Financing Database**
21. Means AR, Ajjampur SSR, Bailey R, Galactionova K, Gwayi-Chore MC, Halliday K, Ibikounle M, Juvekar S, Kalua K, Kang G, et al: **Evaluating the sustainability, scalability, and replicability of an STH transmission interruption intervention: The DeWorm3 implementation science protocol.** *PLoS Negl Trop Dis* 2018, **12:**e0005988.
22. WHO: **Accelerating Work to Overcome the Global Impact of Neglected Tropical Diseases: A Roadmap for Implementation.** Geneva2012.
23. WHO: **Helminth control in school-age children. Second edition. A guide for managers of control programmes.** 2011.
24. Abraham D, Kaliappan SP, Walson JL, Rao Ajjampur SS: **Intervention strategies to reduce the burden of soil-transmitted helminths in India.** *Indian J Med Res* 2018, **147:**533-544.
25. **SurveyCTO mobile data collection platform** [https://www.surveycto.com/]
26. Frick KD: **Microcosting quantity data collection methods.** *Med Care* 2009, **47:**S76-81.
27. Curran GM, Bauer M, Mittman B, Pyne JM, Stetler C: **Effectiveness-implementation Hybrid Designs Combining Elements of Clinical Effectiveness and Implementation Research to Enhance Public Health Impact.** *Medical Care* 2012, **50:**217-226.
28. StataCorp: **Stata Statistical Software: Release 14.** College Station, TX: StataCorp LP; 2015.
29. **Historical Exchange Rates** [https://www.ofx.com/en-au/forex-news/historical-exchange-rates/yearly-average-rates/]
30. Lo NC, Bogoch II, Blackburn BG, Raso G, N'Goran EK, Coulibaly JT, Becker SL, Abrams HB, Utzinger J, Andrews JR: **Comparison of community-wide, integrated mass drug administration strategies for schistosomiasis and soil-transmitted helminthiasis: a cost-effectiveness modelling study.** *Lancet Global Health* 2015, **3:**E629-E638.
31. Leslie J, Garba A, Oliva EB, Barkire A, Tinni AA, Djibo A, Mounkaila I, Fenwick A: **Schistosomiais and Soil-Transmitted Helminth Control in Niger: Cost Effectiveness of School Based and Community Distributed Mass Drug Administration.** *PLOS Neglected Tropical Diseases* 2011, **5:**e1326.
32. Fitzpatrick C, Fleming FM, Madin-Warburton M, Schneider T, Meheus F, Asiedu K, Solomon AW, Montresor A, Biswas G: **Benchmarking the Cost per Person of Mass Treatment for Selected Neglected Tropical Diseases: An Approach Based on Literature Review and Meta-regression with Web-Based Software Application.** *PLoS Negl Trop Dis* 2016, **10:**e0005037.
33. Galactionova K, Velarde M, Silumbe K, Miller J, McDonnell A, Aguas R, Smith TA, Penny MA: **Costing malaria interventions from pilots to elimination programmes.** *Malar J* 2020, **19:**332.

For peer review only - http://bmjopen.bmj.com/site/about/guidelines.xhtml

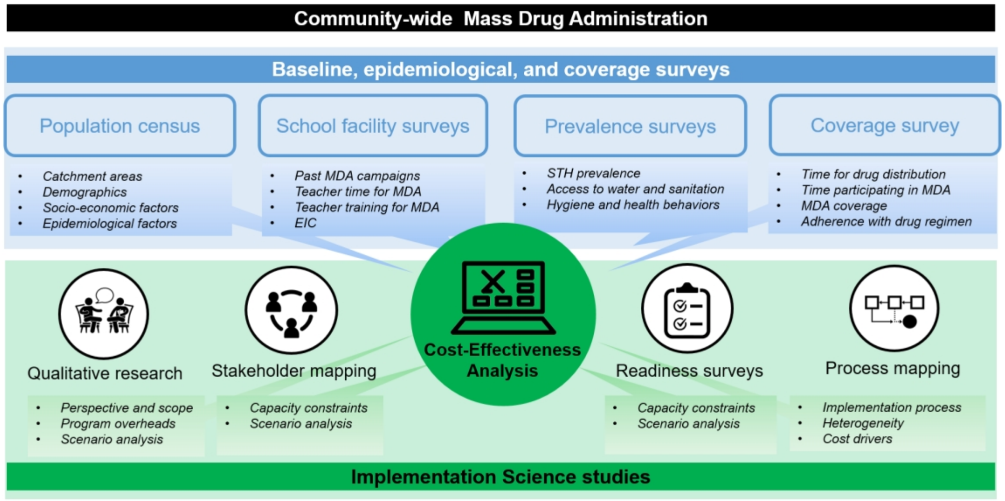

Figure 1. Integration of costing study within the DeWorm3 trial research activities.
The figure illustrates key surveys (top blue panel) and IS studies (bottom green panel) conducted along cMDA in the DeWorm3 trial. It maps cost-effectiveness (to which costing contributes) within the IS research and highlights the contribution of other studies to costing and broader economic research agenda of the project. For further details refer to SI Table S1. Figure adapted from presentation by Means et al "DeWorm3 Project 2017 Annual Site PI Meeting." 2017.

322x163mm (150 x 150 DPI)

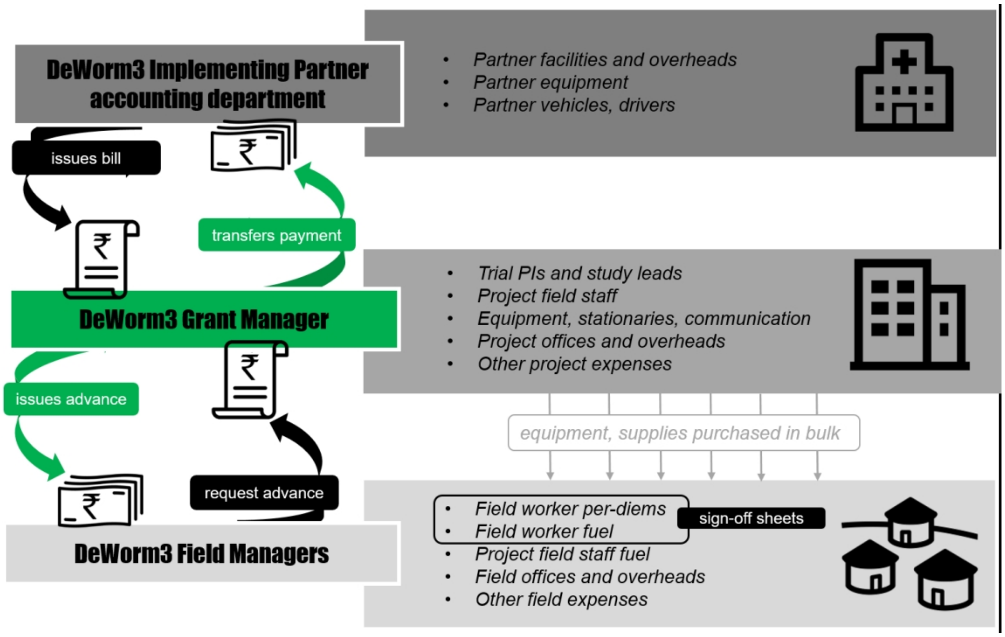

Figure 2. Routine financial reporting at India DeWorm3 trial site
The figure illustrates the flow of funds and financial reporting at India DeWorm3 trial site. Project's grants manager – center green box – is the focal point through which all country funds supporting the trial flow. For each project level the diagram shows staff responsible for dispersing funds (left) and gives examples of some of the resource line items that are purchased/ consumed/ recorded at that level (right panels). Bottom row – field site – additionally highlights itemized tracking of expenditures related to field worker per-diems and fuel (important cost driver of MDA activities).

261x164mm (150 x 150 DPI)

Supplementary Information

Costing interventions in the field: preliminary cost estimates and lessons from an evaluation of community-wide MDA for elimination of STH

Galactionova K[1,2], Sahu M[1,2], Gideon SP[3], Kaliappan SP[3], Morozoff CE[4], Ajjampur SSR[3], Walson J[4], Means AR[4], Tediosi F[1,2]

[1] Swiss Tropical and Public Health Institute, Basel, Switzerland
[2] University of Basel, Basel, Switzerland
[3] Christian Medical College, Vellore, India
[4] University of Washington, Seattle, Washington, USA

Corresponding author:

Katya Galactionova
Socinstrasse 57,
4051 Basel,
Switzerland
e.galactionova@unibas.ch

For peer review only

Box S1. Key terms and definitions

Financial costs: These costs represent actual expenditure on goods and services purchased. Costs are described in terms of how much money has been paid for the resources used in the project or service.

Economic costs: Economic costs, on the other hand, include in addition to financial also costs forgone by using a resource in a particular way. These economic or opportunity costs recognize and value the cost of using resources, as these resources are then unavailable for productive use elsewhere.

Fixed costs: Also referred to as capital costs, indirect costs or overheads are expenses which do no depend with the quantity of output or services delivered by the project. These include rent, equipment lease payments, some wages and salaries.

Variable costs: Are costs that change in proportion to the output or services delivered by the projects. These include supplies, food, fee for service.

Resource category: In the Tool inputs required for implementation of STH MDA-related activities are broadly categorized into *Running costs* and *Activity costs*. *Running costs* refer to resources essential to setting up and running day-to-day operations of the trial. These inputs are employed in a range of trial activities (i.e. shared costs), are retained for the duration of the trial, and often represent a one-time investment (i.e. capital costs or start-up costs). *Activity costs*, on the other hand, refer to resources essential to implementation of specific activities, incremental to those identified under running costs. These inputs are acquired for a specific activity, are retained for the duration of the activity, and often represent recurrent purchases (i.e. require purchase each time the activity is implemented).

Activity: Throughout the trial a variety of activities will be implemented to plan for, deliver, and assess STH MDA. The Tool covers only activities implemented by the DeWorm3 trial team; these include implementation of MDA and related activities in the intervention arm of the trial and activities related to census, monitoring and evaluation, and surveillance in the control arm. Table1 above details the list of activities by implementing partner and trial arm.

Sub-activity: Each activity in turn is divided into sub-activities that represent specific steps or functions necessary to conclude the respective activity. For example “Stool sample collection” is a sub-activity within the “Cross-sectional survey” activity.

Cost classification: Within each sub-activity resources are further grouped into one of seven input classifications; the categories are defined in Table 2 below.

Resource line item: This is a single cost in the Tool. All line items will be associated with a description, cost classification, sub-activity, activity, and resource category.

For peer review only

For peer review only - http://bmjopen.bmj.com/site/about/guidelines.xhtml

Table S1. DeWorm3 trial activities

Activity	Study year (Y)	Tool	Sample	Primary focus	Contribution to economic analyses
Study area census	Y1	Survey CTO	Study area	Enumeration of residents in study area, socio-demographic information, GPS coordinates of dwellings	• Age<br>• School attendance<br>• Population size<br>• Occupation<br>• Asset ownership, dwelling<br>• WASH<br>• Catchment area<br>• Distance to health facility
School facility surveys	Y1-Y5	Survey CTO	Study area	WASH, school enrollment and attendance rates of children	• MDA conducted at school<br>• Time teacher spend helping with the drug delivery<br>• Teacher trained for school de-worming in past 6 months
Cross-sectional prevalence surveys	Y1, Y4, Y5	Kato-Katz, qPCR	500 (1000 for end-line) individuals per cluster	STH prevalence and intensity, DNA	• Deworming in past year<br>• Deworming source<br>• WASH
Longitudinal prevalence surveys	Y1-Y5	Kato-Katz, qPCR	150 individuals per cluster	STH prevalence, intensity, re-infection, DNA	• Time (minutes) CDD spent at the house on the treatment day<br>• Time spent participate in the treatment day, including time waiting for CDD
cMDA	Y1-Y3	Survey CTO	Intervention clusters	MDA coverage	
Coverage surveys	Y1-Y3	Urine test	50 households per cluster	MDA coverage	• Health workers bring medicines or other treatments by the house (last month)<br>• Health worker left tables for intake later<br>• Number of tablets swallowed<br>• Tablets swallowed while the health worker present<br>• Awareness of drug distribution visit<br>• Change to routine to participate in the treatment<br>• Time (minutes) CDD spent at the house on the treatment day<br>• Time spent participate in the treatment day, including time waiting for CDD

For further details on the study protocol refer to Asbjornsdottir et al PLOS 2018

For peer review only

Table S2 Activity Table: cMDA-related activities at the India site in the first trial cycle

Nr	Activity	Sub-activity	Trial arm	Implementation	Resource use
1	Drug procurement and supply chain	Procurement	*Both*	*- Drugs for both arms procured centrally by MoH as part of NTD program procurement<br>- DW3 is part of the national bulk purchase of donated drugs through WHO*	*- GSK donating 200,000 doses to the national NTD program for the trial<br>- Annual procurement<br>- Albendazole testing*
		Storage	*Both*	*DW3 trial drugs are stored at central CMC office with A/C (not paid by DW3)*	*Space requirements for 1 round MDA: 1-2 cupboards to keep 25 medium size boxes of tablets (6ft * 4 ft); total space needed for all rounds MDA: 10ft*10ft*
		Transportation	*Both*	*Grants manager travelled to Mumbai to the Government Medical Depot to take the drugs and arrange courier for shipment to DW3 office in Vellore. From Vellore, drugs were taken to sites using DW3 project vehicle.*	*- Grants manager flight to/from Mumbai, per-diems, accommodation<br>- Courier charges Mumbai to Vellore<br>- Fuel Vellore to field sites*
2	Community-wide MDA round 1 *(implemented as mop-up 1 week after SBD)*	Community sensitization	*Int*	*- Implemented right after NDD<br>- Central DW3 team adapts government developed materials to the trial<br>- State health authorities and local social scientists provide input to IEC adaptation through meetings at central level<br>- ASHA FOs and FSs workers organize and run village meetings*	
		Recruitment of CDDs	*Int*	*- 2 days<br>- 164 total CDDs recruited: 32 were already available, 132 were scouted*	*-Staff time:<br>TC: 1 day<br>FM: 2 days<br>FS (11): 4 days<br>FOs (all): 2 days<br>- Transportation allowance: field staff 3Rs/km<br>-Hired vehicle to transport recruiters*
		Training and Piloting	*Int*	*- CDDs 3 days:<br>CDDS/ASHA workers are trained by DW3 Medical Officer at cluster level.<br>- FOs FS 4 days:<br>FOs (109) and FS (11) trained on forms by TC, DM, TL<br>- AE/SAE training for Medical Team 1 day:<br>MO gave training to 2 DW3 study nurses*	*- CDD/ASHAs received travel reimbursement (100 rupees) and refreshments<br>-Travel reimbursement and refreshments for CDD/ASHA workers<br>- No refreshments provided, only regular transport allowance for FOs, FS<br>- No transport allowance for AE/SAE trainings<br>-Fuel for vehicle to transport TC, DM, TL to field (4 days)*
		MDA	*Int*	*- DW3 FS supervise MDA<br>- MDA delivered by CDDs accompanied by FOs, all by foot<br>- 2 MOs and 2 nurses support in the field with AE<br>- 164 CDDs/ASHA workers total, working on average 5 days each for a total of 26 days worked.*	*-108 FOs<br>-1 CDD at a time per FW<br>-Village health nurse hired for 15 days to sensitize, administer and mop-up MDA<br>- CDDs/ASHA workers are paid an honorarium (200 Rs/day) + mobile allowance (50 Rs/round) for a total of 173,400Rs<br>- 44 VHNs , SHNs, CHNs paid 500 Rs/round for a total of 22,000Rs<br>- Office supply costs including printing of logs<br>- Job aids, bags, posters, banners, flip charts*

		Post-MDA coverage survey	*Int*	*- Includes 1 day of training for FOs by TC and DM<br>- Data processing done centrally by local data team*	*- Staff time: DM,TC,FOs<br>- Office supply costs including printing of logs*
		Mop-up MDA	*Int*	*- FOs and FS did mop-up and a few CDDs/ASHA*	*No allowances given to CDDs/ASHA*
3	Drug procurement and supply chain	Procurement	*Int*	*- Drugs for both arms procured centrally by MoH as part of NTD program procurement<br>- DW3 is part of the national bulk indent of donated drugs through WHO*	*-GSK donating 200,000 doses to the national NTD program for the trial<br>- Albendazole quality control testing*
		Storage	*Int*	*DW3 trial drugs are stored at central CMC office with A/C (not paid by DW3)*	*Space requirements for 1 round MDA: 1-2 cupboards to keep 25 medium size boxes of tablets (6ft * 4 ft); total space needed for all rounds MDA: 10ft*10ft*
		Transportation	*Int*	*Grants Manager travelled to Mumbai to the Government Medical Depot to collect the drugs and arrange courier for shipment to DW3 office in Vellore. From Vellore, drugs were taken to sites using DW3 project vehicle.*	*- Grants Manager flight to/from Mumbai, per-diems, accommodation<br>- Courier charges Mumbai to Vellore<br>- Fuel Vellore to field sites*
4	Community-wide MDA round 2	Community sensitization	*Both*	*- CAB meetings 1 day: TC, PI, FM, MO, IS (4), FS (8), 12 CAB members<br>- Community sensitization meetings 11 days: FM and 3 FS and 5 FO went to 82 villages conducted 82 meetings (5 persons per meeting)<br>- Joined meetings organized by Grama Sabha to sensitize village heads and local community leaders<br>- FS/FO travel by personal bikes*	*- Staff time: PI,TC,MO,FM, FS<br>- Food expense for community-level CAB meetings<br>- BMOs 1000Rs honorarium, others (schoolteachers, block superintendent, opinion leaders etc) 500Rs, Food 237 Rs/person<br>- FS/FOs receive transport allowance 3 Rs per km<br>- Fuel for DW3 project staff (CMC vehicle)*
		Training and Piloting	*Int*	*- CDDs/ASHA 2 days: CDDS/ASHA workers are trained by DW3 MO at cluster level<br>- 4 days: FOs (60) and FS (10) trained on forms by TC, DM, TL.<br>-AE/SAE training for Medical Team 1 day: MO gave training to 2 DW3 study nurses*	*- CDD/ASHAs received travel reimbursement (100 rupees) and refreshments<br>- No refreshments provided, only regular transport allowance for FO, FS trainings<br>- No transport allowance for AE trainings<br>-Fuel for vehicle to transport TC, DM, TL to field (4 days)*
		MDA	*Int*	*-DW3 field supervisors supervise MDA<br>-MDA delivered by CDDs accompanied by FO (70), all on foot<br>- 2 Medical officers and 2 nurses support in the field with adverse event<br>-114 CDDs/ASHA workers total, working on average 5 days each for a total of 12 days worked.*	*- CDDs/ASHA workers are paid an honorarium (200 Rs/day) + mobile allowance (50 Rs/round) for a total of Rs188,650<br>- Office supply costs including printing of logs<br>- Job aids, bags, posters, banners, flip charts*
		Post-MDA coverage survey	*Both*	*- Includes 1 day of training for FOs by TC and DM<br>- Data processing done centrally and by local data team*	*- Staff time: DM,TC, FOs, DM<br>- Printing charges<br>- Office supply costs including printing of logs*
		Mop-up MDA	*Int*	*- FOs and FS did mop-up (and a few CDDs/ASHA)*	*No allowances given to CDDs/ ASHA workers*

Int= Intervention arm; MoH= Ministry of Health; MoE= Ministry of Education; CMC= Christian Medical College, Vellore; TC= trial coordinator; FM= field manager; FS= field supervisor; FO= field officer; DM= data manager

For peer review only - http://bmjopen.bmj.com/site/about/guidelines.xhtml

Table S3 Cost classification of inputs in the DeWorm3 costing tool

No	Input classification	Define	Examples
1	Drugs	Anti-helminthic medication distributed during MDA campaigns and used to treat adults with moderate to high intensity infections	Albendazole
2	Wages and per-diems	Fixed regular payment earned for work or services, per-diem allowances, and any other monetary awards paid to personnel involved in the trial	Per-diems
3	Vehicles and overheads	Means of transportation, including hire and transportation allowance, vehicle maintenance, fuel	Transportation allowance, fuel allowance
4	Equipment and overheads	Supplies and tools that last more than a year and have a unit cost at or above 100 USD; including maintenance and overheads	Mobile phones, Microscope, Equipment maintenance
4	Buildings and overheads	Facilities occupied by DeWorm3 trial and supporting teams	Rent payment, utilities incurred at DeWorm3 site offices
5	Materials and supplies	Commodities required to support an operation or activity	SIM cards, office supplies
6	Other	All other inputs outside of the either of the above categories	Consumables (i.e. food and drinks)

For peer review only

For peer review only - http://bmjopen.bmj.com/site/about/guidelines.xhtml

Table S4 Allocation of resource line items to trial activities and categorization of resource line-items into cMDA and research-related and allocation rules of management overheads to trial activities

Resource category	Resource line items	Activity	Activity allocation rule	cMDA	Research
Staff wages and per-diems	Trial PI and core trial staff	Program management	Permanent staff, direct allocation based on % time working on trial	Yes	Yes
	Field staff	Activity	Temporary staff, direct allocation based on number of days supporting activity	Yes	Yes
	Laboratory staff	Prevalence surveys	Permanent staff, direct allocation based on % time working on trial; temporary staff, direct allocation based on number of days supporting activity	No	Yes
	Data staff	Program management	100%	No	Yes
Vehicles and overheads	Vehicles owned	Program management	100%	Yes	Yes
	Vehicles rented	Program management	100%	Yes	Yes
	Transportation related expenses of field and other project staff	Activity	Direct allocation based on number of days supporting activity	Yes	Yes
Equipment and overheads	Office equipment	Program management	100%	Yes	Yes
	Mobile phones	Census	100%	No	Yes
	Field worker kits	Activity	100%	Yes	Yes
	Laboratory equipment	Prevalence survey	100%	No	Yes
Buildings and overheads	Central	Program management	100%	Yes	Yes
	Field offices	Program management	100%	Yes	Yes
Materials and supplies	Stationaries and other materials	Activity	Direct allocation based on number of days supporting activity	Yes	Yes
Other	Trial insurance	Program management	100%	No	Yes

For peer review only - http://bmjopen.bmj.com/site/about/guidelines.xhtml

Table S5 Average annual financial costs of STH cMDA* implementation in India DeWorm3 trial site, USD 2018

Sub-activity	Start-up**		Program management		Drug testing and distribution to sites		CDD recruitment		Community sensitization		Training		cMDA		Coverage survey		Mop-up	
Cost category	US$	%	US$	%	US$	%	US$	%	US$	%	US$	%	US$	%	US$	%	US$	%
Wages and per-diems***	2,027	94.3	26,520	63.4	0	0.0	1,199	57.2	11,283	50.7	4,322	55.1	18,666	66.4	3,232	58.1	2,388	57.8
Buildings and overheads	80	3.7	2,139	5.1	0	0.0	0	0.0	0	0.0	0	0.0	0	0.0	0	0.0	0	0.0
Equipment and overheads	0	0.0	857	2.0	0	0.0	0	0.0	7	0.0	0	0.0	0	0.0	0	0.0	0	0.0
Transportation	22	1.0	7,562	18.1	553	87.0	858	41.0	7,590	34.1	3,360	42.8	7,709	27.4	2,215	39.8	1,665	40.3
Communication	7	0.3	3,378	8.1	0	0.0	0	0.0	0	0.0	0	0.0	205	0.7	0	0.0	0	0.0
Materials and supplies	11	0.5	1,384	3.3	0	0.0	38	1.8	3,078	13.8	135	1.7	395	1.4	120	2.2	75	1.8
Other	2	0.1	0	0.0	83	13.0	0	0.0	309	1.4	26	0.3	1,126	4.0	0	0.0	0	0.0
Total	2,149	100.0	41,840	100.0	636	100.0	2,095	100.0	22,267	100.0	7,843	100.0	28,101	100.0	5,567	100.0	4,128	100.0

*cMDA was implemented as a mop-up following NDD campaign that targeted school-aged children; ** Start-up activities annualized over the duration of the trial (5 years). *** Wages and per-diems include project staff, field workers, and incentives to CDDs. Costs were converted to USD using average annual exchange rate over the study period (1 INR=0.01462 USD) [26]. STH= Soil-Transmitted Helminth infections; cMDA= community-wide Mass Drug Administration; CDD= Community Drug Distributor.

For peer review only

For peer review only - http://bmjopen.bmj.com/site/about/guidelines.xhtml

Table S6 First year financial costs of STH cMDA* implementation in India DeWorm3 trial site, USD 2018: adjusted base rate

	Total (US$)			Average cost Profile (%)	Average cost per person treated per round (US$)	Average cost per Capita per round (US$)
MDA round	1	2	1+2	1+2	1+2	1+2
**TOTAL**	62,819	57,672	120,491	100.0	1.20	0.88
**TOTAL incremental****	4,829	6,577	11,406	9.5	0.11	0.08
Start-up activities***	1,094	1,055	2,149	1.8	0.02	0.02
Program management	21,300	20,540	41,840	34.7	0.42	0.31
Drug testing and distribution to site	304	331	636	0.5	0.01	0.00
CDD recruitment	1,160	1,119	2,279	1.9	0.02	0.02
Community sensitization	13,972	10,235	24,207	20.1	0.24	0.18
CDD bags and job aids	555	428	983	0.8	0.01	0.01
Banners, posters	969	969	1,937	1.6	0.02	0.01
Training	4,826	3,222	8,048	6.7	0.08	0.06
Travel allowance for CDDs	240	170	409	0.3	0.00	0.00
Refreshments for CDDs	10	17	26	0.0	0.00	0.00
cMDA	14,495	16,065	30,560	25.4	0.30	0.22
CDD incentives	2,632	4,577	7,210	6	0.07	0.05
CDD mobile allowance	120	85	205	0.2	0.00	0.00
Coverage survey	3,404	2,803	6,207	5.2	0.06	0.05
Mop-up	2,262	2,302	4,564	3.8	0.05	0.03

*cMDA was implemented as a mop-up following NDD campaign that targeted school-aged children; **Incremental costs represent a subset of rows highlighted in *cursive*, see text for details. *** Start-up activities annualized over the duration of the trial (5 years). Grey shaded rows are a subset of the higher level activity grouping. Total number of people treated in first round of cMDA (cMDA1) was 51’320 (site total population 68’442); in second round (cMDA2) – 49’488 (site total population 68’460); total treated over the two rounds (cMDA1+cMDA2) was 100'808 (total population 136'902). Costs were converted to USD using average annual exchange rate over the study period (1 INR=0.01462 USD) [27]. STH= Soil-Transmitted Helminth infections; cMDA= community-wide Mass Drug Administration; CDD= Community Drug Distributor.

For peer review only - http://bmjopen.bmj.com/site/about/guidelines.xhtml

File 1. DeWorm3 Costing Tool data collection templates

Module1: Planning

A1.1 Planning

	Number of days						
Description	Recruitment	Procurement	Planning trial activities	Developing IEC materials	Developing training materials	Remarks	Source
I	III	IV	V	VI	VII	VIII	IX

A1.2 Pre-trial sensitization

A1.2.1 Pre-trial sensitization meetings

Level of meeting	Location	Type of meeting space	Length of meeting (days)	Number of meetings	Date(s)
National					
State					
District					
Block					
Public health center					
Health sub center					
Cluster					

For peer review only

For peer review only - http://bmjopen.bmj.com/site/about/guidelines.xhtml

A1.2.2 Pre-trial sensitization meetings attendees

	Sensitization meeting							
Description	Level of meeting	Trial staff (Yes/No)	Transportation allowance per meeting (LCU)	Daily lodging allowance (LCU)	Daily food allowance (LCU)	Other allowance (LCU)	Remarks	Source
I	II	III	IV	V	VI	VII	VIII	IX

A1.2.3 Pre-trial sensitization meetings attendees

	Sensitization meeting							
Description	Level of meeting	Trial staff (Yes/No)	Transportation allowance per meeting (LCU)	Daily lodging allowance (LCU)	Daily food allowance (LCU)	Other allowance (LCU)	Remarks	Source
I	II	III	IV	V	VI	VII	VIII	IX

For peer review only

Module2: Shared resources and program overheads

P2.1 Wages and per-diems

Description	Employed by trial (Yes/No)	Working on trial (%)	Number of staff units (units)	Monthly wage (LCU)	Other monthly benefits (LCU)	Daily per-diem rate (LCU)	Remarks	Source
I	II	III	XVI	XVII	XVIII	XIX	XX	XXI

P2.2 Vehicles and overheads

Description	Purchased by trial (Yes/ No)	Used by trial (%)	Number of vehicles (units)	Make	Year of productio n	Year of purchas e	Price (LCU)	Average annual overhead costs (LCU)	Average annual maintena nce costs (LCU)	Remarks	Source
I	II	III	IV	V	VI	VII	VIII	IX	X	XI	XII

For peer review only - http://bmjopen.bmj.com/site/about/guidelines.xhtml

P2.2.1 Reference fuel prices

Fuel	Price units	Price per unit (LCU)	Km per L	L per km	Remarks

P2.2.2 Reference distances

Location	Distance units	Distance (one way)

P2.2.3 Other transportation related expenses

Description	Monthly travel expenditures (LCU)										
	Month, year	Month, year	Month, year	Month, year	Month, year	Month, year	Month, year	Month, year	Month, year	Month, year	Month, year

For peer review only - http://bmjopen.bmj.com/site/about/guidelines.xhtml

P2.3 Equipment and overheads

Description	Purchased by DeWorm3 (Yes/ No)	Used by DeWorm3 (%)	Number of units (units)	Make	Year of production	Year of purchase	Total Price (LCU)	Import duties and other charges (LCU)	Average annual overhead costs (LCU)	Average annual maintenance costs (LCU)	Remarks	Source
I	III	IV	V	VI	VII	VIII	IX	X	XI	XII	XIII	XIV

P2.3.1 Reference office furnishings prices and calculations

Furnishings	Unit price (LCU)	Number of units (units)	Total (LCU)

P2.4 Buildings and overheads

Description	Rented by DeWorm3 (Yes/ No)	Used by DeWorm3 (%)	Number of facilities (units)	Monthly rent per sq foot (LCU)	Size (sq feet)	Average monthly overheads (LCU)	Total including monthly overheads (LCU)	Remarks	Source
I	III	IV	V	VI	VII	VIII	IX	X	XI

For peer review only - http://bmjopen.bmj.com/site/about/guidelines.xhtml

P2.5 Materials and supplies

Description	Number of units (units)	Rate per unit (LCU)	Rate units	Remarks	Source
I	II	III	IV	V	VI

P2.5.1 Other materials and supplies

Description	Monthly materials and supplies expenditures (LCU)										
	Month, year	Month, year	Month, year	Month, year	Month, year	Month, year	Month, year	Month, year	Month, year	Month, year	Month, year

P2.6 Other

Description	Purchased by DeWorm3 (Yes/ No)	Used by DeWorm3 (%)	Number of units (units)	Rate per unit (LCU)	Rate units	Other charges (LCU)	Total Charges (LCU)	Remarks	Source
I	II	III	*IV*	V	VI	VII	VIII	IX	X

For peer review only - http://bmjopen.bmj.com/site/about/guidelines.xhtml

Module3: Activity

A3.1 Sub-activity

Cost classification	Description	Number of units (units)	Units	Insurance (LCU)	Freight (LCU)	Travel charges (LCU)	Handling fee (LCU)	Total (LCU)	Remarks	Source
I	II	III	IV	V	VI	VII	VIII	IX	X	XI

For peer review only - http://bmjopen.bmj.com/site/about/guidelines.xhtml

For peer review only

For peer review only - http://bmjopen.bmj.com/site/about/guidelines.xhtml

BMJ Open

Costing interventions in the field: preliminary cost estimates and lessons learned from an evaluation of community-wide Mass Drug Administration for elimination of Soil-Transmitted Helminths in the DeWorm3 trial

Journal:	*BMJ Open*
Manuscript ID	bmjopen-2021-049734.R1
Article Type:	Original research
Date Submitted by the Author:	11-May-2021
Complete List of Authors:	Galactionova, Katya; Swiss Tropical and Public Health Institute; University of Basel<br>Sahu, Maitreyi; Swiss Tropical and Public Health Institute; University of Basel<br>Gideon, Samuel; Christian Medical College Vellore<br>PUTHUPALAYAM KALIAPPAN, SARAVANAKUMAR; Christian Medical College Vellore<br>Morozoff, Chloe; University of Washington<br>Ajjampur, Sitara; Christian Medical College Vellore<br>Walson, Judd; University of Washington<br>Rubin Means, Arianna; University of Washington<br>Tediosi, Fabrizio; Swiss Tropical and Public Health Institute; University of Basel
**Primary Subject Heading**:	Global health
Secondary Subject Heading:	Infectious diseases
Keywords:	HEALTH ECONOMICS, Health policy < HEALTH SERVICES ADMINISTRATION & MANAGEMENT, Infection control < INFECTIOUS DISEASES, Public health < INFECTIOUS DISEASES, INFECTIOUS DISEASES

SCHOLARONE™
Manuscripts

I, the Submitting Author has the right to grant and does grant on behalf of all authors of the Work (as defined in the below author licence), an exclusive licence and/or a non-exclusive licence for contributions from authors who are: i) UK Crown employees; ii) where BMJ has agreed a CC-BY licence shall apply, and/or iii) in accordance with the terms applicable for US Federal Government officers or employees acting as part of their official duties; on a worldwide, perpetual, irrevocable, royalty-free basis to BMJ Publishing Group Ltd ("BMJ") its licensees and where the relevant Journal is co-owned by BMJ to the co-owners of the Journal, to publish the Work in this journal and any other BMJ products and to exploit all rights, as set out in our licence.

The Submitting Author accepts and understands that any supply made under these terms is made by BMJ to the Submitting Author unless you are acting as an employee on behalf of your employer or a postgraduate student of an affiliated institution which is paying any applicable article publishing charge ("APC") for Open Access articles. Where the Submitting Author wishes to make the Work available on an Open Access basis (and intends to pay the relevant APC), the terms of reuse of such Open Access shall be governed by a Creative Commons licence – details of these licences and which Creative Commons licence will apply to this Work are set out in our licence referred to above.

Other than as permitted in any relevant BMJ Author's Self Archiving Policies, I confirm this Work has not been accepted for publication elsewhere, is not being considered for publication elsewhere and does not duplicate material already published. I confirm all authors consent to publication of this Work and authorise the granting of this licence.

For peer review only

Costing interventions in the field: preliminary cost estimates and lessons learned from an evaluation of community-wide Mass Drug Administration for elimination of Soil-Transmitted Helminths in the DeWorm3 trial

Galactionova K[1,2], Sahu M[1,2], Gideon SP[3], Kaliappan SP[3], Morozoff C[4], Ajjampur SSR[3], Walson J[4], Means AR[4], Tediosi F[1,2]

[1] Swiss Tropical and Public Health Institute, Basel, Switzerland
[2] University of Basel, Basel, Switzerland
[3] Christian Medical College, Vellore, India
[4] University of Washington, Seattle, Washington, USA

Corresponding author:

Katya Galactionova
Socinstrasse 57,
4051 Basel,
Switzerland
e.galactionova@unibas.ch

Word count: 5505

For peer review only

Keywords:

costs, cost data collection, field trial, Soil-Transmitted Helminths, community-wide MDA

For peer review only

For peer review only - http://bmjopen.bmj.com/site/about/guidelines.xhtml

Author Information:

Katya Galactionova	e.galactionova@unibas.ch
Mita Sahu	msahu@uw.edu
Samuel Paul Gideon	sampaulgide@gmail.com
Saravanakumar Puthupalayam Kaliappan	epipks@gmail.com
Chloe Morozoff	chloemor@uw.edu
Sitara Swarna Rao Ajjampur	sitararao@cmcvellore.ac.in
Judd Walson	walson@uw.edu
Arianna Means	aerubin@uw.edu
Fabrizio Tediosi	fabrizio.tediosi@swisstph.ch

For peer review only

For peer review only - http://bmjopen.bmj.com/site/about/guidelines.xhtml

Abstract

Objective: To present a costing study integrated within the DeWorm3 multi-country field trial of community-wide Mass Drug Administration (cMDA) for elimination of Soil-transmitted Helminths.

Design: Tailored data collection instruments covering resource use, expenditure, and operational details were developed for each site. These were populated alongside field activities by on-site staff. Data quality control and validation processes were established. Programmed routines were used to clean, standardize, and analyze data to derive costs of cMDA and supportive activities.

Setting: Field site and collaborating research institutions.

Primary and secondary outcome measures: A strategy for costing interventions in parallel with field activities was discussed. Interim estimates of cMDA costs obtained with the strategy were presented for one of the trial sites.

Results:

The study demonstrated that it was both feasible and advantageous to collect data alongside field activities. Practical decisions on implementing the strategy and the trade-offs involved varied by site; trialists and local partners were key to tailoring data collection to the technical and operational realities in the field. The strategy capitalized on the established processes for routine financial reporting at sites, benefitted from high recall, and gathered operational insight that facilitated interpretation of the estimates derived. The methodology produced granular costs that aligned with the literature and allowed exploration of relevant scenarios. In the first year of the trial, net of drugs, the incremental financial cost of extending deworming of school-aged children to the whole community in India site averaged 1.14 (USD, 2018) per person per round. A hypothesized at-scale routine implementation scenario yielded a much lower estimate of 0.11 USD per person treated per round.

Conclusions: We showed that costing interventions alongside field activities offers unique opportunities for collecting rich data to inform policy toward optimizing health interventions and for facilitating transfer of economic evidence from the field to the program.

For peer review only - http://bmjopen.bmj.com/site/about/guidelines.xhtml

Article Summary

Strengths and limitations of this study

- Resource use data were collected by on-site trial teams alongside field activities.
- This ensured high recall, supported collaboration between trial teams, and grounded costing in operational insight.
- Separating data collection from the analysis, facilitated tailoring of tools without undermining consistency of the estimates between sites.
- While comprehensive in the scope of resources evaluated, the methodology was labor intensive to implement and required expertise to derive costs from the data collated.

For peer review only

Introduction

Field trials conducted outside of clinical settings generate important evidence for making public health decisions and optimizing health programs. They indicate what the impact of efficacious interventions might be under real-world conditions, within representative target populations, and with implementation processes that aim to inform routine programmatic activities[1]. Increasingly, economic outcomes, including costs and cost-effectiveness, are evaluated within community-based research studies to generate evidence regarding sustainability and scalability of tested interventions[2-6].

While methodological guidance on economic evaluation and valuation of costs has been clearly outlined[7-10], guidance on practical aspects of cost data collection in the field is relatively scarce[11]. Normative documents, including the recently released Reference Case for Global Health Costing[10], cover some aspects of cost data collection including the perspective to take, the level at which to conduct costing, and offer guidance on cost classification categories to adopt for presenting results of the study. However, apart from a brief overview of approaches for planning a costing exercise and potential data sources, these guidelines are limited in their description of strategies for integrating costing studies within field trials. A number of technical documents, targeted primarily at program managers, offer guidance on budgeting of health interventions[9, 12-15]; these program-specific guidelines emphasize retrospective evaluation of interventions directed by costing templates or interactive tools. Guidelines for cost data collection along clinical trials, on the other hand, support ongoing costing activities[16, 17]. Although primarily aimed at developed country contexts, these documents discuss a number of considerations relevant for field evaluations such as the need to isolate trial-driven resource use, the importance of prioritizing data collection toward resource line items that are most likely to change the policy decision (i.e. if costing is to support cost-effectiveness analyses), and integration of costing within the routine trial reporting. Finally, guidelines on costing health interventions in low- and middle-income countries address specific challenges that arise due to poor data availability and limited economic expertise in these countries[18, 19]. The guidelines further highlight the trade-offs between accuracy and data availability and suggest use of mixed methodologies (i.e. gross costing along with bottom-up approaches) to tackle these limitations.

Integrating costing within a multi-country field trial raises basic practical questions not adequately addressed by this literature. How to assign roles for cost data collection and analysis, when to collect the data and for how long, what level of detail is appropriate and can be feasibly accommodated in the context of a field study, which tools to use, how to balance specificity of sites with the need for consistent comparison of costs between sites,

For peer review only - http://bmjopen.bmj.com/site/about/guidelines.xhtml

how best to accommodate multiple economic endpoints and so on. Choices made by researchers to address these questions have important implications for the cost estimates derived and for their relevance for policy decisions the trial ultimately aims to inform. Somewhat surprisingly, there has been remarkably little exchange between practitioners on their experience with different strategies for costing interventions in the field[19, 20].

In this paper, we present our strategy for incorporating a costing study within the DeWorm3 project (ClinicalTrials.gov Identifier NCT03014167) – a community-based cluster-randomized trial testing the feasibility of interrupting transmission of soil-transmitted helminth (STH) infections in India, Benin, and Malawi[2, 21]. This project is funded by the Bill & Melinda Gates Foundation through a grant awarded to the Natural History Museum (NHM). The DeWorm3 interventions consist of bi-annual community-wide mass drug administration (cMDA) targeting eligible individuals of all ages compared to standard of care deworming of school-age children (SAC). The trial is conducted in focal geographic areas with a population between 100,000 to 140,000 residing in each country site. CMDA is deployed for three years (six rounds) followed by two years of monitoring for recrudescence. The project aims to generate evidence for local and global stakeholders on optimal strategies for control and elimination of STH, including multi-level factors influencing treatment coverage[21].

Although the trial and its evaluation, including costing, are ongoing, we share our early experience with the methodology to stimulate exchange among practitioners on the design and implementation of costing studies in the field. We discuss here some of the practical choices made and ways in which these decisions were motivated by the aims of the project, the infrastructure, and the implementation of the trial interventions in specific sites. We report preliminary cost estimates for the India trial site and reflect on the challenges encountered in implementing our costing strategy, the ways in which we addressed these, and highlight the lessons learned for future costing studies operating in similar contexts.

Methods

Interventions

The current global strategy for STH control relies on MDA in SAC[22]. The frequency and targeting of MDA campaigns are determined by the prevalence of infection in SAC with more frequent treatment where prevalence is high[23]. The Indian STH program distributes MDA with Albendazole to pre-SAC and SAC children via a bi-annual national mass campaign called the National Deworming Day (NDD)[24]. Drugs are administered during school days by teachers under the supervision of the local medical staff and the Ministry of Health (MoH).

For peer review only - http://bmjopen.bmj.com/site/about/guidelines.xhtml

cMDA programs for STH control, on the other hand, target eligible community members of all ages. In India cMDA in DeWorm3 intervention clusters started a week after NDD; drugs were administered to all individuals who were not treated in schools (i.e. adults and out-of-school children). Prior to drug distribution, the DeWorm3 staff sensitized the community by distributing IEC materials, designed based on the NDD campaign with additional artwork depicting the treatment of whole communities, and by making public announcements. Community Drug Distributors (CDDs) were recruited from community volunteers and Accredited Social Health Activist (ASHA) workers. CDDs and project field officers supporting drug distribution were trained by the DeWorm3 team on consent seeking, drug administration, and data collection. A medical team was trained on adverse event monitoring. During the campaign rounds, CDDs were accompanied by project field officers and supervised by project staff. CDDs were paid a daily per-diem and received a mobile data package for each campaign round. Additionally, health facilities received an annual honorarium payment for health center staff that supported the project on site. MDA coverage was assessed in both intervention and control clusters by field officers using mobile devices equipped with SurveyCTO[25]. Data were uploaded to a central database and analyzed by the trial data management team. Mop-up drug distributions were conducted in all clusters in all rounds. A detailed description of operational activities and resource use supporting cMDA is presented in SI Tables S1-2.

Study purpose, perspective and scope

The purpose of this study was to present our strategy for integrating costing into the DeWorm3 field trial[21]. To inform the broader aims of the project with respect to the cost-effectiveness of cMDA, the societal perspective was taken in the costing evaluation, and an assessment of both financial (most relevant for NTD programs and short-term planning) and economic costs (most relevant for MOH and global stakeholders) was undertaken (See SI Box S1 for key terms). Costs were evaluated around an operational intervention model following the micro-costing approach[26]. This methodology, while effort-intensive, grounded the evaluation in the implementation process and yielded granular data to inform modeling of cMDA modalities and contexts beyond those observed in the trial.

Integration of costing within the DeWorm3 project

Integrating a costing study within a clinical trial required deciding who will collect the data, when, and for how long. The DeWorm3 project included local institutions with strong epidemiological and clinical field research expertise (Table 1). Capitalizing on the interest of these organizations to develop capacity for economic evaluation, in-country teams were designated to lead cost data collection using standardized tools and with support from off-site

For peer review only - http://bmjopen.bmj.com/site/about/guidelines.xhtml

health economics research groups. Ensuring country ownership was central to DeWorm3 design, as the project aims to facilitate transfer of knowledge from the field to support STH programs and policies in countries hosting the trial.

Table 1. Implementation of DeWorm3 trial at study sites

Location	Grant-holding organization	Implementing organization and partners	National Standard of Care STH Control Strategy (age target)	Community-wide STH MDA	Primary costing point person(s)
Come Commune, Benin	Institut de Recherche Clinique pour Développement	- Institut de Recherche Clinique du Benin<br>- Institut de Recherche Clinique pour Développement<br>- Ministry of Health, Benin	School-based MDA (5-14 years old)	Bi-annual community-wide MDA in all ages	Accountant, Assistant accountant, IS lead
Tamil Nadu State, India	Christian Medical College, Vellore	- Christian Medical College, Vellore<br>- Ministry of Health and Family Welfare, India	School-based MDA and National Deworming days (1-19 years old)	Bi-annual community-wide MDA in all ages; as a mop-up following NDD	Grant manager, Trial coordinator
Mangochi District, Malawi	Blantyre Institute for Community Outreach	- Blantyre Institute for Community Outreach<br>- London School of Tropical Medicine and Hygiene<br>- Ministry of Health and Education, Malawi	School-based MDA and National Deworming days (1-14 years old)	Bi-annual community-wide MDA in all ages	Accountant, Field accountant, Field manager

DeWorm3 is a hybrid trial, which incorporates implementation and operational questions into a clinical trial design[27]. Since costs are an implementation outcome, economic analyses were considered under the umbrella of implementation science (IS) research (Figure1). The IS studies collected data on operational processes, health systems and policy contexts, and community experience with cMDA. Thus, the data collection for the costing study could narrowly focus on direct financial and economic costs of cMDA to the project.

Figure1

The costing study covered all field activities (Figure1, black and blue panels). This removed the need for cost data collection point persons at each site to decide which expenditures to

For peer review only - http://bmjopen.bmj.com/site/about/guidelines.xhtml

record and allowed for a comprehensive assessment of resources that contributed to cMDA coverage and, ultimately, intervention effectiveness. Moreover, by tracking resources beyond operational activities immediately supporting cMDA, the study collated inputs for modeling costs of programmatic components such as surveillance and monitoring that are part of routine NTD control (i.e. population census and prevalence surveys).

Cost data were collected over two years (four MDA rounds) in order to capture improvements in efficiency of the program as teams gained operational experience deploying the intervention over multiple rounds.

Integration of costing within the trial sites

The key feature of our study design is the delineation of responsibilities for data collection and valuation between the collaborators. Costing teams at each site were responsible for identifying resources used to support implementation of trial activities, extracting information on their respective prices and quantities, and recording these data using standardized data collection instruments. The off-site economics team, on the other hand, was responsible for triangulating these data to value resources in economic terms.

We conducted several rounds of training and formative meetings with site-designated costing point persons and other supporting staff. Several instruments, described briefly in Table 2, were developed to guide cost data collection. These tools included an Excel-based Costing Tool, an Activity Table, and an Activity Calendar. The Costing Tool detailed by activity quantities, prices, and costs of the resources used, while the Activity Table described implementation of activities costed in the field covering additional resources for which no expenditures were incurred. Data entry templates from the Costing Tool and an example of a filled-out Activity Table for India site are shared in the Supplementary Information file (SI File S1 and Table S2).

Table 2 DeWorm3 instruments for collecting inputs to economic analysis

Costing instrument	Type of cost	Content	Responsible Person	Location of responsibl e person
Activity Table*	Financial and Opportunity	Description of operational activities and sub-activities, number of project staff and other resources used, number of days	Grant Manager, Trial coordinator	Field
Activity Calendar		Start, end dates, and duration of operational activities	Field manager, Trial coordinator	Field

For peer review only - http://bmjopen.bmj.com/site/about/guidelines.xhtml

For peer review only

Costing Tool	Financial	Resource line items, corresponding prices, quantities, and expenditure recorded by sub-activity; separate modules for start-up and implementation	Grant manager, Trial coordinator	Site
Spend Report	Financial	Trial expenditures	Project financial manager	External
Central Expenditure Report	Financial	List of prices and quantities of items procured at the central level	Project financial manager	External

*An example of an Activity Table detailing a subset of trial activities implemented related to cMDA is enclosed in SI, Table S2

In the Costing Tool, resources were tracked differently by stage of implementation, specifically distinguishing between planning and other pre-trial activities (i.e. start-up) and activities taking place during the trial itself. For the start-up phase, sites reported the number of meetings, time dedicated to planning, sensitization of country stakeholders, and development of training and sensitization materials for the trial. In the implementation stage, in addition to resource lists and quantities, information was collected also on prices, expenditure and other details relevant to economic valuation (e.g. year of purchase for capital items). The implementation modules of the Costing Tool further distinguished between resources that contributed to multiple trial activities (shared resources) such as trial PI, office building, vehicles, etc. from resources that contributed to a single activity. Shared resources were recorded in the Costing Tool at the start of the trial and were reviewed for completeness at each round of data collection. Thus, for each trial activity, only the incremental expenditures (i.e. those additional to program overheads and capital) were recorded by sites, thus minimizing the effort and facilitating allocation of expenditure data.

Institutional differences and differences in technical capacity between sites required tailoring of the data collection strategy to each partner. Where reporting was centralized, one costing point person was designated with cost data collection, where different staff were responsible for dispensing funds for specific operational activities or resources (i.e. fuel or bulk purchases of supplies) multiple staff assumed responsibility for compiling cost data at their respective levels. During piloting visits, the economics team, working together with in-country partners, mapped the costing strategy into sites' routine financial reporting, tailored to the site the costing instruments developed, and updated standard operating procedures that outlined roles, responsibilities, and reporting timelines for costing at the site.

For peer review only - http://bmjopen.bmj.com/site/about/guidelines.xhtml

As an illustrative example, Figure2 shows the flow of funds and financial reporting at DeWorm3 India site. The site designated their grants manager to lead cost data collection. Guided by the Costing Tool, the grants manager extracted information from receipts originating from the project's central offices, the Christian Medical College (CMC), Vellore which hosted the trial, and the field sites. Expenditures that were incurred outside of the trial site (i.e. bulk purchases of commodities and supplies by NHM DeWorm3 central team) were sourced and added to the Costing Tool in the analysis stage. Integrated within the trial operations, the costing study benefitted from site's established financial reporting processes, quality control, and oversight by partner institutions.

Figure2

From costing inputs to an estimate of intervention costs

Separating cost data collection from valuation of resources meant that the bulk of data cleaning, categorization, consistency checks, and validation happened during data quality and assurance checks at the central level. Entries in the Costing Tool were first evaluated for completeness and then for consistency by comparing expenditures and resource use against activities implemented as detailed in the Activity Table. During data quality checks, we standardized descriptions of resource line items, cost categories, and activities to allow comparisons within and between different activities at each site. We checked for duplicate entries, ensured that the same resources were priced at the same rate, and that similar activities yielded comparable costs. Where inconsistencies were identified, site teams provided additional operational details and clarifications to resolve the issue, and brainstormed on whether adjustments to the analysis were needed to reflect the implementation.

Once cleaned, we applied standardized programming routines, implemented in Stata[28], to convert these data into estimates of cMDA and trial costs. Specifically:

1. **Start-up costs:** We retrospectively estimated costs of planning and other pre-trial activities for which no expenditure data were recorded. In the Costing Tool these were described according to the type (i.e. sensitization meetings, micro-planning) and intensity of activities conducted (i.e. the number of meetings). We used information from other sections of the Costing Tool (i.e. project staff wages, transportation) to value the respective resource use via direct allocation. Start-up costs were treated as capital items and were annualized over the fiver year duration of the trial.

For peer review only - http://bmjopen.bmj.com/site/about/guidelines.xhtml

2. **Activity costs:** Costs of trial operational activities were obtained by summing costs of the resource line items recorded in the Costing Tool and adding on shared resources and program overheads. Resource line items were valued according to their respective cost category (SI Table S3) applying, where appropriate, annualization and adjusting for percentage used for the trial to estimate the value of these resources for the first cMDA cycle.

3. **Shared costs:** Shared resources that could be allocated to specific activities and sub-activities such as laboratory equipment were allocated accordingly, while the rest (primarily research and management staff and overheads) were grouped under "Program management" cost category (SI Table S4). Resources that contributed to multiple activities, such as some transportation-related expenses were allocated based on the number of activity days.

To derive cost of cMDA, we isolated cMDA-related expenditures from all other trial expenditures. For the most part, the distinction between intervention and research costs was tied to activity or sub-activity category (i.e. expenditures related to training of CDDs supporting MDA were categorized as cMDA-related). If, however, an activity or a resource category contributed to both cMDA and research activities, in some sites, was the case for training of field workers, costs were allocated based on the ratio of cMDA days to total trial days. For some shared resources, costs were allocated multiple times: first to the respective activity and then to cMDA or the trial (SI Table S4). Total cost of cMDA was then obtained by summing over sub-activities supporting drug administration, and adding on cMDA-attributable share of planning and program costs.

Differences in implementation of the costing approach between sites required further tailoring of these standardized valuation and allocation routines. In India, for instance, compensation of field staff and some other field expenditures (i.e. materials and supplies, some fuel and transportation costs) were tracked on a monthly, rather than weekly or daily, basis. These monthly expenditure totals often covered multiple activities each requiring a varying number of field staff. To allocate these, we first calculated a daily equivalent value for each expenditure type and, where possible, staff category. Specifically, monthly expenditures were summed over the 12-month cycle within which the two rounds of cMDA were delivered and divided by the total number of staff, compensated each month times 20.5 (the average number of working days per month according to India team). We then mapped these monthly expenditure categories into trial activities based on Activity Table, flagging activities to which a specific staff or resource category contributed. Finally, activity totals were obtained by

For peer review only - http://bmjopen.bmj.com/site/about/guidelines.xhtml

multiplying the daily equivalent value for each expenditure type by the number of staff and the number of days over which the respective activity was conducted. To the extent that multiple activities, including those research-related, were conducted simultaneously by the field staff this strategy might have overstated total cMDA-attributable spending.

Costs of cMDA in the DeWorm3 India site

To support a critical assessment of the study design presented, we share preliminary costs for the first year of cMDA in the DeWorm3 India site. These are interim findings that only integrate financial expenditures, final cost estimates from DeWorm3 are forthcoming. The incremental financial costs of cMDA were summarized by key operational activities, including start-up and program overheads. We additionally considered how our results from the trial might translate to costs under routine programmatic implementation by Indian NDD program.

Patient and Public Involvement

No patient involved.

Results

Table 3 presents interim results for two rounds of cMDA and supportive activities conducted at the India DeWorm3 site between October 2017 and September 2018. In the first year, the cost averaged about $1.14 per person treated per round. While the unit costs were virtually identical between the two rounds, there were differences in the number of field staff (lower in the second round) and the number of implementation days for sensitization, training, and drug distribution campaign (higher in the second round). Drug administration in the community accounted for over a quarter of total intervention cost (row “cMDA”, Table 3). As drugs were donated, cMDA costs were primarily driven by personnel: wages of field staff and incentives to CDDs accounted of about 70% of total costs (SI Table S5). The second-largest cost component was “Program management and overheads” (33% of total cost). This cost category covers wages of core trial personnel and expenses related to transportation for supportive supervision or other activities conducted by this management cadre. Program overheads also include data processing and data management costs (i.e. related to registration of households for MDA).

For peer review only - http://bmjopen.bmj.com/site/about/guidelines.xhtml

Table 3 Interim results from first year of STH cMDA* implementation in India DeWorm3 site: financial costsUSD 2018

	Total (US$)			Average cost profile (%)	Average cost per person treated per round (US$)	Average cost per capita per round (US$)
MDA round	1	2	1+2	1+2	1+2	1+2
TOTAL	60,320	54,305	114,626	100.0	1.14	0.84
*TOTAL incremental***	*4,829*	*6,577*	*11,406*	*10.0*	*0.11*	*0.08*
Start-up activities***	1,094	1,055	2,149	1.9	0.02	0.02
Program management	21,300	20,540	41,840	36.5	0.42	0.31
Drug testing and distribution to site	304	331	636	0.6	0.01	0.00
CDD recruitment	1,066	1,028	2,095	1.8	0.02	0.02
Community sensitization	12,924	9,343	22,267	19.4	0.22	0.16
CDD bags and job aids	555	428	983	0.9	0.01	0.01
Banners, posters	969	969	1,937	1.7	0.02	0.01
Training	5,003	2,840	7,843	6.8	0.08	0.06
Travel allowance for CDDs	240	170	409	0.4	0.00	0.00
Refreshments for CDDs	10	17	26	0.0	0.00	0.00
cMDA	13,442	14,659	28,101	24.5	0.28	0.21
CDD incentives	2,632	4,577	7,210	6.3	0.07	0.05
CDD mobile allowance	120	85	205	0.2	0.00	0.00
Coverage survey	3,116	2,451	5,567	4.9	0.06	0.04
Mop-up	2,070	2,058	4,128	3.6	0.04	0.03

*cMDA was implemented as a mop-up following NDD campaign that targeted school-aged children; **Incremental costs represent a subset of rows highlighted in *cursive*, see text for details. *** Start-up activities annualized over the duration of the trial (5 years). Grey shaded rows are a subset of the higher level activity grouping. Total number of people treated in first round of cMDA (cMDA1) was 51'320 (site total population 68'442); in second round (cMDA2) – 49'488 (site total population 68'460); total treated over the two rounds (cMDA1+cMDA2) was 100'808 (total population 136'902). Costs were converted to USD using average annual exchange rate over the study period (1 INR=0.01462 USD) [29]. STH= Soil-Transmitted Helminth infections; cMDA= community-wide Mass Drug Administration; CDD= Community Drug Distributor.

For peer review only - http://bmjopen.bmj.com/site/about/guidelines.xhtml

Due to the large contribution of field staff to the overall cost of cMDA, the estimates were highly sensitive to decisions on how these resources were quantified and valued. The estimates presented in Table 3 above were obtained by applying a fixed monthly base rate to the number of staff and activity days to derive total cost. In practice, though, there was some variation around the amount paid to field staff. At the India site, field workers were borrowed from other studies to assist in drug distribution. These additional staff were compensated at their regular rate which was below the rate paid in the DeWorm3 project. Moreover, during the campaign, field staff also worked on weekends and through holidays earning overtime. To better capture the value of field staff we re-ran the analysis applying an adjustment weight to the base wage. The site staff-level weight was calculated as a ratio of the respective monthly base rate to monthly equivalent rate derived from site expenditure data. It varied from 0.9 to 2.5 between staff categories: nearly identical to the monthly base rate for field officers (weights ranging between 0.9 and 1.01) and substantially above the base rate for field managers and supervisors indicating overtime. One weight was then applied to all sub-activities that started in a given month thus inflating base wages depending on the total expenditure incurred in a given month. After this rate adjustment the average cost per person treated per round increased to $1.20 (SI Table S6).

Arguably, from the perspective of the program the primary interest is in the incremental resource needs for reaching adults and children not treated under the current NDD deworming campaign. Thus, the programmatic relevance of the totals reported for the trial above and some of the resource line items costed warrant further consideration. For example, it could be suggested that planning, program management, and community sensitization activities would not necessarily change to accommodate the broader age target for cMDA. Provided there is spare capacity in the system, costs related to drugs, drug storage and distribution, and also coverage surveys and mop-up, would scale linearly with the number of people covered. The incremental costs, compared to the current SAC-targeted strategy, would then be incurred for recruitment, training, and incentives of/to CDD's (See Table 3 rows highlighted in cursive). This scenario adds up to a significantly lower cost estimate per person treated of $0.11, compared to $1.14 reported from the perspective of the trial. Further analyses with input from the program are needed to appropriately contextualize these findings.

Recognizing that comparisons of costs between studies are often limited due to differences in the objectives of respective evaluations, scope of resources costed as well as methodological choices made by analysts[10], these comparisons, nonetheless, provide additional support to the methodology described here. We found that the cost estimates

For peer review only - http://bmjopen.bmj.com/site/about/guidelines.xhtml

derived align reasonably well with cMDA costs reported elsewhere. For instance, a recent multi-site study from African region reported an average of $1.75 per person treated per round of community-wide MDA treatment[30]. A lower estimate of $0.65[1] per person treated was reported over a 2 year program in 4 districts in Niger, this is about half the estimate of drug delivery for SAC in the same study[31]. The scale of the program: 300,000 to over 450,000 people treated in Niger compared to just under 70,000 in the DeWorm3 India site explains some of the difference in costs between the two studies. Another estimate comes from a meta-regression of MDA costs for NTDs; net of community volunteers and drugs, the estimate averaged $0.40 (range $0.02 to $2.90) per person per round[32], which is comparable to $0.58 in this study when we exclude payments to CDDs and field officers.

Discussion

The costing strategy presented here was developed in close collaboration with trialists both at and off-site that helped us adapt it to the technical and operational realities at sites. The methodology capitalized on the immediacy of resource use on the ground, the operational expertise, and established processes for routine financial reporting within the implementing organizations. Integrating costing within the DeWorm3 trial allowed us to build on other research studies conducted concomitantly to get a fuller appreciation of resource use related to cMDA. Implementing this strategy required a multitude of practical decisions that were necessarily context specific and entailled trade-offs between precision and effort. We carefully consider these below to inform choices analysts make when designing costing exercises in similar settings. We hope to encourage exchange among practitioners on their experience with different methodologies for costing interventions in the field.

Balancing flexibility and consistency in designing the strategy

One of the key challenges in conducting a multi-country cost analysis is in balancing the need for flexibility to accommodate the trial and intervention features in a given setting with the need for consistency and standardization of cost estimates across the trial sites. Our reliance on the micro-costing methodology and use of multiple instruments to detail operational activities on the ground, including resource inputs, helped capture these differences. Piloting of the tools, and their tailoring to and integration into routine financial reporting further ensured that robust estimates could be derived despite differences in sites' contextual features. Adapting data collection to sites, however, did complicate the analysis. We could ensure consistent valuation of resources by relying on programmed routines that

[1] Niger GDP deflator applied to 2005 estimate of $0.46 reported in the study[10]

For peer review only - http://bmjopen.bmj.com/site/about/guidelines.xhtml

fixed *how* resources were categorized, priced, annualized, and allocated to the intervention across sites.

Harnessing synergies and fostering collaborations within the project

The complexity of the DeWorm3 project posed a number of own challenges for the design and implementation of the costing study. With many research activities conducted alongside the intervention (e.g. annual census, prevalence surveys), developing a costing strategy required coordination between the many project partners on and off-site. From decisions on who will lead the cost data collection to when to pilot the costing tools, to how best to monitor quality of the data collected - all had to be agreed upon between multiple parties. The communication overhead was minimized by integrating the costing study under the broader IS research agenda; additionally, reporting was further streamlined by centralized tracking of costing outputs by the IS team which formally tied costing deliverables to funding transfers to sites.

While in the design stages of the project, we could identify synergies between studies, differences in the timing of data collection and processing meant that we could not fully avoid some duplication of effort. For instance, Activity Table compiled by field staff (i.e. field manager or trial coordinator) at each site collated some of the same information as IS process mapping exercises. Activity Table filled out by the field staff during each trial activity was immediately available to the costing point person at the site and the economic teams at the submission of costing instruments; the IS outputs, on the other hand, populated with data from multiple study clusters, required time for data collection and processing before these could be integrated. At the same time, the overlap between data collection instruments allowed for triangulation of data in the analysis stage, which further strengthened the quality of the evidence.

The success of our costing strategy further relied on close cooperation between the different study leads at the site. Efficient communication and management within the implementing agency meant that it was possible for the designated field staff and costing point persons to validate resource allocation to specific activities and reflect those appropriately in the costing instruments. When sites were limited for time in collecting or reviewing costs, higher levels of aggregation of expenditure data were recorded.

Challenges and opportunities in country-led cost data collection

We found that we could support site data collection efforts best when our guidance was grounded in an understanding of operational details of interventions being evaluated and the

For peer review only - http://bmjopen.bmj.com/site/about/guidelines.xhtml

specific contexts in which the evaluation was conducted. To this end, delaying site visits and piloting of tools until field activities have commenced appeared to have been one of the critical choices we made in implementing the strategy. This ensured that both financing channels and routine reporting at sites were established by the time of the visit and we could map these together with costing leads to guide where and how best to collect the data.

Our initial guidance on selecting the focal person for cost data collection was an on-site team member that was primarily field-based and had a part-time duty to populate cost data collection instruments. However, due to the sensitivities of financial reporting, particularly of salaries, it was challenging to designate anyone other than accounting staff to collate economic data. This naturally imposed a substantial burden on accountants already fully engaged in their primary duties. To make it possible for an accountant, removed from the field, to lead cost data collection it was necessary to develop additional instruments (i.e. Activity Table) and to facilitate a closer involvement of field staff with the costing study (i.e. via Activity Table, Activity Calendar) than originally anticipated. On the other hand, these additional tools enabled unique insights into the implementation of interventions on the ground and facilitated quality control and subsequent analyses of the data collected.

Routine financial reporting at sites offered some unanticipated opportunities for cost data collection. For example, in Malawi, weekly financial advances were made by an off-site accountant to the field team which were reconciled the following week against a filled-out Expenditure Ledger. The latter, organized in an Excel table, itemized field expenses detailing prices, quantities along with a brief description for each entry. Both the frequency of recording and the level of detail at which expenditures were described in the Ledger made it easy to integrate this site-developed tool into the costing strategy.

Some of the project research studies were conducted either concomitantly with or relying on resources that also supported cMDA. Allocation of shared resources is an area that requires value judgments which are often difficult for those new to costing. There are several aspects of our strategy that made these choices less arbitrary. First, extending the scope of the evaluation to all trial activities (including research-related) removed the need for local teams to decide which activities or resources to track. Second, capital resource line items and other resources that contributed to multiple activities like program staff were explicitly identified and tracked separately in the Costing Tool, thus focusing data collection on tracking incremental expenditures for each activity and leaving allocation of shared resources to the economics team.

For peer review only - http://bmjopen.bmj.com/site/about/guidelines.xhtml

Separating data collection and valuation of resources ensured that inconsistencies in data entry could be resolved in the analysis stage. This feature proved advantageous as, despite multiple points of validation at the field site, entries in the Costing Tool were often misallocated between activities. These challenges highlight limitations of pre-populated costing tools that collate aggregated expenditures recorded by field teams into an estimate of unit costs. We were able to resolve these errors by triangulating information across the costing instruments and by comparing entries between the trial sites; being able to follow-up with teams shortly after an activity was completed to validate any corrections to the data ensured high recall.

Sites also struggled with the level of detail for cost data collection. Modifying internal reporting templates to include information on prices, quantities, and activities to which an expenditure/ resource line item contributed made it easier for sites to capture these data. However, it required more time to fill out these documents. Where there were multiple financially responsible persons the volume of receipts, particularly those attesting to petit-cash spending or other low-level expenditures, created a substantial hurdle in recording and transferring these data into costing tools.

Sites voiced difficulties mapping expenditures in the Costing Tool into their internal accounting documents. To allow sites to double-check expenditure totals and to ensure that expenditures were accurately and comprehensively reported in both systems we included an additional column in the Tool for sites' internal transfer number. These linked resource line items in the Tool to the internal financial tranche that paid for it, which further improved reporting.

We found that our approach worked best in sites where there was an established and lean process for financial reporting within the implementing organization. Where bureaucracy was heavy, for instance, when reporting was required for multiple funders each with own accounting system there was low capacity to support data collection for the costing study. Multiple staff were required to facilitate the process and to transfer financial and resource use data into a format that could be used for the economic analysis.

Relevance to programs

The cost estimates of cMDA implementation presented here are only the first step in the economic analysis. Most immediately, the scope of the evaluation will expand to consider the full economic costs of the program and broaden the perspective to include indirect costs to the community and the project by triangulating data from surveys conducted along the trial

For peer review only - http://bmjopen.bmj.com/site/about/guidelines.xhtml

(Figure1). Informed with IS research, costing will further explore cost drivers and heterogeneity in cost of cMDA within and between sites. In India, the analysis will compare costs between two study sub-sites that differ in their population, socio-cultural and economic characteristics, and geography. IS research will further help identify the incremental resource use for current and alternative cMDA implementation scenarios to feed back to programs.

The operational model for cMDA implementation in the trial detailed in Activity Table forms the basis for an assessment of the delivery modality within the project to what delivery might look like when scaled up by the program. Using the data collected, we can begin to isolate aspects of intervention design that are trial-driven and update these with assumptions reflecting operational realities of the program. When presenting cost estimates for the trial we hypothesized the potential scope of incremental resources that might be relevant to India NTD program, the resulting estimate, describing primarily incentives to CDDs and community sensitization aids was drastically different than the one describing the full scope of resources that supported cMDA implementation in the trial (i.e. $0.11 compared to $1.14 per person treated per round). The methodology thus facilitates careful interpretation of the costs derived and supports effective communication of findings to the national program recognizing the many ways in which the scalable model might differ from the one piloted and the likely cost implications of these differences[17, 33].

Conclusion

Field research produces important insights about effectiveness of interventions in the community. The programmatic relevance of field trials can be further strengthened by integrating costing within the trial study design. We showed here that collecting cost data along field activities is both feasible and immediately informed by the implementation of interventions on the ground. The strategy presented offers unique opportunities for collecting granular data to support a broad range of policy questions toward optimizing health interventions and facilitating transfer of economic evidence from the field to the program.

For peer review only - http://bmjopen.bmj.com/site/about/guidelines.xhtml

Figure legends

Figure 1. Integration of costing study within the DeWorm3 trial research activities.

The figure illustrates key surveys (top blue panel) and IS studies (bottom green panel) conducted along cMDA in the DeWorm3 trial. It maps cost-effectiveness (to which costing contributes) within the IS research and highlights the contribution of other studies to costing and broader economic research agenda of the project. For further details refer to SI Table S1. Figure adapted from presentation by Means et al "DeWorm3 Project 2017 Annual Site PI Meeting." 2017.

Figure 2. Routine financial reporting at India DeWorm3 trial site.

The figure illustrates the flow of funds and financial reporting at India DeWorm3 trial site. Project's grants manager – center green box – is the focal point through which all country funds supporting the trial flow. For each project level the diagram shows staff responsible for dispersing funds (left) and gives examples of some of the resource line items that are purchased/ consumed/ recorded at that level (right panels). Bottom row – field site – additionally highlights itemized tracking of expenditures related to field worker per-diems and fuel (important cost driver of MDA activities).

Author contribution: KG, ARM, FT designed data collection tools; KG piloted data collection tools; SPG, SPK collected data and contributed to design of data collection tools; CM, MS conducted data queries; KG developed data analysis and validation routines; KG, CM analyzed the data; KG wrote the first draft; KG, ARM, CM wrote the manuscript. MS, FT provided critical inputs and edits to the manuscript. KG, MS, SPG, SPK, CM, SSRA, JW, ARM, FT reviewed and approved the final version of the manuscript for publication.

Conflict of interest: The authors declare no conflict of interest.

Funding: This project was funded by a sub-contract from the Natural History Museum grant from the Bill and Melinda Gates foundation OPP1129535.

Acknowledgements: We would like to acknowledge in-country teams and off-site project partners who generously contributed their experience and insight to the design of the costing study and who continue to support cost data collection within the DeWorm3 trial. Specifically, Wongani Lungu, Chikondi Chikotichalera, Roselyn Hara, Euripide Avokpaho, Nadege Aicheou, Monrenike Bada, James Simwanza, David Chinyanya, Rajesh Kumar Rajendiran, Chinnaduraipandi Paulsamy, Elodie Yard, Stefan Witek-McManus, Katherine E Halliday, Hugo Legge, Leanne Doran, William E Oswald, Fabian Schär, Marie-Claire Gwayi-Chore, Mira Emmanuel-Fabua, Iain Gardiner, Moudachirou Ibikounle, Khumbo Kalua, Adrian JF Luty, and Robin Bailey. We thank the community advisory boards at each site for supporting this study.

Data sharing: All data relevant to the study are included in the article or uploaded as supplementary information. No additional data available.

For peer review only - http://bmjopen.bmj.com/site/about/guidelines.xhtml

References

1. In *Field Trials of Health Interventions: A Toolbox.* Edited by rd, Smith PG, Morrow RH, Ross DA. Oxford (UK)2015: *Wellcome Trust-Funded Monographs and Book Chapters*].
2. Asbjornsdottir KH, Ajjampur SSR, Anderson RM, Bailey R, Gardiner I, Halliday KE, Ibikounle M, Kalua K, Kang G, Littlewood DTJ, et al: **Assessing the feasibility of interrupting the transmission of soil-transmitted helminths through mass drug administration: The DeWorm3 cluster randomized trial protocol.** *PLoS Negl Trop Dis* 2018, **12:**e0006166.
3. Pullan RL, Halliday KE, Oswald WE, McHaro C, Beaumont E, Kepha S, Witek-McManus S, Gichuki PM, Allen E, Drake T, et al: **Effects, equity, and cost of school-based and community-wide treatment strategies for soil-transmitted helminths in Kenya: a cluster-randomised controlled trial.** *Lancet* 2019, **393:**2039-2050.
4. Pitt C, Ndiaye M, Conteh L, Sy O, Hadj Ba E, Cisse B, Gomis JF, Gaye O, Ndiaye JL, Milligan PJ: **Large-scale delivery of seasonal malaria chemoprevention to children under 10 in Senegal: an economic analysis.** *Health Policy Plan* 2017, **32:**1256-1266.
5. Means AR, Kemp CG, Gwayi-Chore MC, Gimbel S, Soi C, Sherr K, Wagenaar BH, Wasserheit JN, Weiner BJ: **Evaluating and optimizing the consolidated framework for implementation research (CFIR) for use in low- and middle-income countries: a systematic review.** *Implementation Science* 2020, **15**.
6. Proctor E, Silmere H, Raghavan R, Hovmand P, Aarons G, Bunger A, Griffey R, Hensley M: **Outcomes for implementation research: conceptual distinctions, measurement challenges, and research agenda.** *Adm Policy Ment Health* 2011, **38:**65-76.
7. Walker D: **Cost and cost-effectiveness guidelines: which ones to use?** *Health Policy and Planning* 2001, **16:**113-121.
8. Drummond MF, Sculpher MJ, Torrance GW, O`Brien BJ, Stoddart GL: *Methods for the economic evaluation of health care programmes.* 3 edn. Oxford, United Kingdom: Oxford University Press; 2005.
9. Phillips MA, Mills A, Dye C: **Guidelines for cost-effectiveness analysis of vector control.** In *PEEM Guidelines Series 3*. Geneva, Switzerland1993.
10. Vassall A, Sweeney S, Kahn J, Gomez GB, Bollinger L, Marseille E, Herzel B, DeCormier Plosky W, Cunnama L, Sinanovic E, et al: **Reference Case for Global Health Costing.** 2016.
11. Crowell V, Levin A, Galactionova K, Tediosi F: **Costing and budgeting for malaria service delivery.** Basel, Switzerland: Swiss Tropical and Public Health Institute; 2013.
12. Creese A, Parker D: **Cost analysis in primary health care: a training manual for program managers.** Geneva, Switzerland1994.
13. UNAIDS: **Workbook for collection of Cost Information of HIV Services.** 2011.
14. UNAIDS: **Costing Guidelines for HIV Prevention Strategies.** Geneva: Joint United Nations Programme on HIV/AIDS; 2000.
15. Immunization VaB: **WHO-UNICEF guidelines for developing comprehensive multi-year plan (cMYP).** 2014.
16. Doshi JA, Glick HA, Polsky D: **Analyses of cost data in economic evaluations conducted alongside randomized controlled trials.** *Value in Health* 2006, **9:**334-340.
17. O'Sullivan AK, Thompson D, Drummond MF: **Collection of health-economic data alongside clinical trials: Is there a future for piggyback evaluations?** *Value in Health* 2005, **8:**67-79.
18. Hendriks ME, Kundu P, Boers AC, Bolarinwa OA, te Pas MJ, Akande TM, Agbede K, Gomez GB, Redekop WK, Schultsz C, Tan SS: **Step-by-step guideline for disease-

For peer review only - http://bmjopen.bmj.com/site/about/guidelines.xhtml

specific costing studies in low-and middle-income countries: a mixed methodology. *Global Health Action* 2014, **7**.
19. Batura N, Pulkki-Brannstrom AM, Agrawal P, Bagra A, Haghparast-Bidgoli H, Bozzani F, Colbourn T, Greco G, Hossain T, Sinha R, et al: **Collecting and analysing cost data for complex public health trials: reflections on practice.** *Global Health Action* 2014, **7**.
20. **The cMYP Immunization Financing Database**
21. Means AR, Ajjampur SSR, Bailey R, Galactionova K, Gwayi-Chore MC, Halliday K, Ibikounle M, Juvekar S, Kalua K, Kang G, et al: **Evaluating the sustainability, scalability, and replicability of an STH transmission interruption intervention: The DeWorm3 implementation science protocol.** *PLoS Negl Trop Dis* 2018, **12:**e0005988.
22. WHO: **Accelerating Work to Overcome the Global Impact of Neglected Tropical Diseases: A Roadmap for Implementation.** Geneva2012.
23. WHO: **Helminth control in school-age children. Second edition. A guide for managers of control programmes.** 2011.
24. Abraham D, Kaliappan SP, Walson JL, Rao Ajjampur SS: **Intervention strategies to reduce the burden of soil-transmitted helminths in India.** *Indian J Med Res* 2018, **147:**533-544.
25. **SurveyCTO mobile data collection platform** [https://www.surveycto.com/]
26. Frick KD: **Microcosting quantity data collection methods.** *Med Care* 2009, **47:**S76-81.
27. Curran GM, Bauer M, Mittman B, Pyne JM, Stetler C: **Effectiveness-implementation Hybrid Designs Combining Elements of Clinical Effectiveness and Implementation Research to Enhance Public Health Impact.** *Medical Care* 2012, **50:**217-226.
28. StataCorp: **Stata Statistical Software: Release 14.** College Station, TX: StataCorp LP; 2015.
29. **Historical Exchange Rates** [https://www.ofx.com/en-au/forex-news/historical-exchange-rates/yearly-average-rates/]
30. Lo NC, Bogoch II, Blackburn BG, Raso G, N'Goran EK, Coulibaly JT, Becker SL, Abrams HB, Utzinger J, Andrews JR: **Comparison of community-wide, integrated mass drug administration strategies for schistosomiasis and soil-transmitted helminthiasis: a cost-effectiveness modelling study.** *Lancet Global Health* 2015, **3:**E629-E638.
31. Leslie J, Garba A, Oliva EB, Barkire A, Tinni AA, Djibo A, Mounkaila I, Fenwick A: **Schistosomiais and Soil-Transmitted Helminth Control in Niger: Cost Effectiveness of School Based and Community Distributed Mass Drug Administration.** *PLOS Neglected Tropical Diseases* 2011, **5:**e1326.
32. Fitzpatrick C, Fleming FM, Madin-Warburton M, Schneider T, Meheus F, Asiedu K, Solomon AW, Montresor A, Biswas G: **Benchmarking the Cost per Person of Mass Treatment for Selected Neglected Tropical Diseases: An Approach Based on Literature Review and Meta-regression with Web-Based Software Application.** *PLoS Negl Trop Dis* 2016, **10:**e0005037.
33. Galactionova K, Velarde M, Silumbe K, Miller J, McDonnell A, Aguas R, Smith TA, Penny MA: **Costing malaria interventions from pilots to elimination programmes.** *Malar J* 2020, **19:**332.

For peer review only

For peer review only - http://bmjopen.bmj.com/site/about/guidelines.xhtml

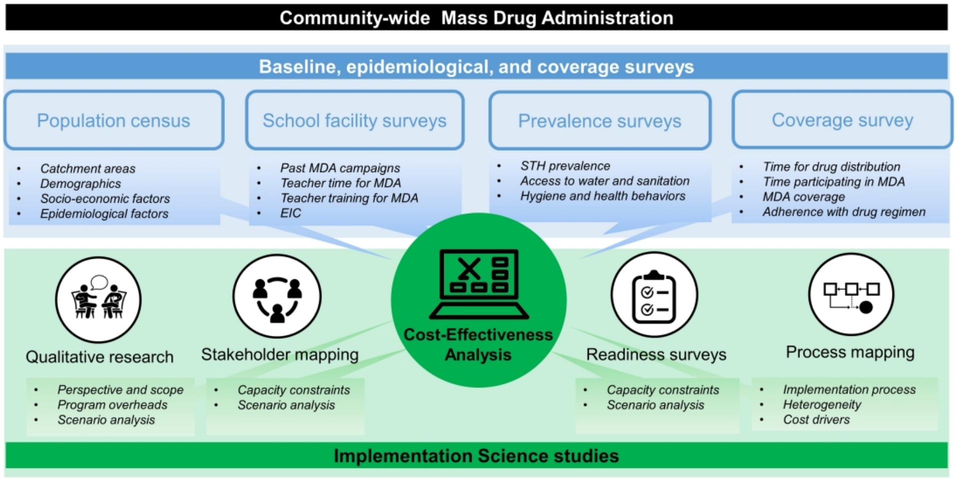

Figure 1. Integration of costing study within the DeWorm3 trial research activities.
The figure illustrates key surveys (top blue panel) and IS studies (bottom green panel) conducted along cMDA in the DeWorm3 trial. It maps cost-effectiveness (to which costing contributes) within the IS research and highlights the contribution of other studies to costing and broader economic research agenda of the project. For further details refer to SI Table S1. Figure adapted from presentation by Means et al "DeWorm3 Project 2017 Annual Site PI Meeting." 2017.

338x190mm (300 x 300 DPI)

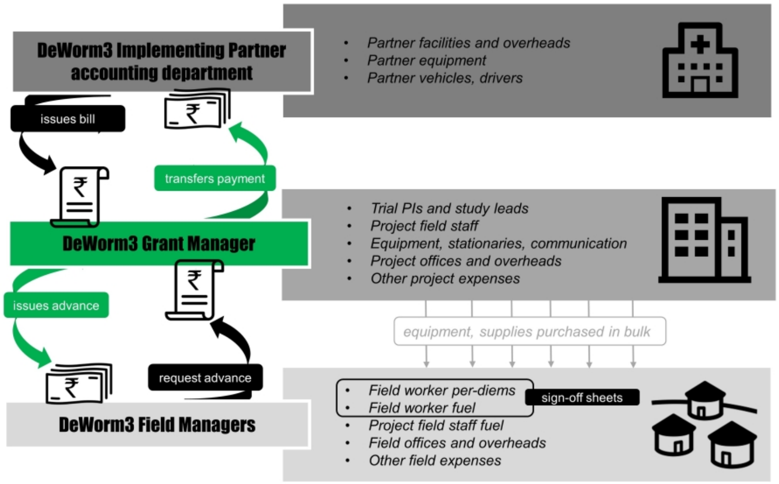

Figure 2. Routine financial reporting at India DeWorm3 trial site.
The figure illustrates the flow of funds and financial reporting at India DeWorm3 trial site. Project's grants manager – center green box – is the focal point through which all country funds supporting the trial flow. For each project level the diagram shows staff responsible for dispersing funds (left) and gives examples of some of the resource line items that are purchased/ consumed/ recorded at that level (right panels). Bottom row – field site – additionally highlights itemized tracking of expenditures related to field worker per-diems and fuel (important cost driver of MDA activities).

338x190mm (300 x 300 DPI)

Supplementary Information

Costing interventions in the field: preliminary cost estimates and lessons from an evaluation of community-wide MDA for elimination of STH

Galactionova K[1,2], Sahu M[1,2], Gideon SP[3], Kaliappan SP[3], Morozoff CE[4], Ajjampur SSR[3], Walson J[4], Means AR[4], Tediosi F[1,2]

[1] Swiss Tropical and Public Health Institute, Basel, Switzerland
[2] University of Basel, Basel, Switzerland
[3] Christian Medical College, Vellore, India
[4] University of Washington, Seattle, Washington, USA

Corresponding author:

Katya Galactionova
Socinstrasse 57,
4051 Basel,
Switzerland
e.galactionova@unibas.ch

For peer review only - http://bmjopen.bmj.com/site/about/guidelines.xhtml

Box S1. Key terms and definitions

Financial costs: These costs represent actual expenditure on goods and services purchased. Costs are described in terms of how much money has been paid for the resources used in the project or service.

Economic costs: Economic costs, on the other hand, include in addition to financial also costs forgone by using a resource in a particular way. These economic or opportunity costs recognize and value the cost of using resources, as these resources are then unavailable for productive use elsewhere.

Fixed costs: Also referred to as capital costs, indirect costs or overheads are expenses which do no depend with the quantity of output or services delivered by the project. These include rent, equipment lease payments, some wages and salaries.

Variable costs: Are costs that change in proportion to the output or services delivered by the projects. These include supplies, food, fee for service.

Resource category: In the Tool inputs required for implementation of STH MDA-related activities are broadly categorized into *Running costs* and *Activity costs*. *Running costs* refer to resources essential to setting up and running day-to-day operations of the trial. These inputs are employed in a range of trial activities (i.e. shared costs), are retained for the duration of the trial, and often represent a one-time investment (i.e. capital costs or start-up costs). *Activity costs*, on the other hand, refer to resources essential to implementation of specific activities, incremental to those identified under running costs. These inputs are acquired for a specific activity, are retained for the duration of the activity, and often represent recurrent purchases (i.e. require purchase each time the activity is implemented).

Activity: Throughout the trial a variety of activities will be implemented to plan for, deliver, and assess STH MDA. The Tool covers only activities implemented by the DeWorm3 trial team; these include implementation of MDA and related activities in the intervention arm of the trial and activities related to census, monitoring and evaluation, and surveillance in the control arm. Table1 above details the list of activities by implementing partner and trial arm.

Sub-activity: Each activity in turn is divided into sub-activities that represent specific steps or functions necessary to conclude the respective activity. For example "Stool sample collection" is a sub-activity within the "Cross-sectional survey" activity.

Cost classification: Within each sub-activity resources are further grouped into one of seven input classifications; the categories are defined in Table 2 below.

Resource line item: This is a single cost in the Tool. All line items will be associated with a description, cost classification, sub-activity, activity, and resource category.

For peer review only

Table S1. DeWorm3 trial activities

Activity	Study year (Y)	Tool	Sample	Primary focus	Contribution to economic analyses
Study area census	Y1	Survey CTO	Study area	Enumeration of residents in study area, socio-demographic information, GPS coordinates of dwellings	• Age<br>• School attendance<br>• Population size<br>• Occupation<br>• Asset ownership, dwelling<br>• WASH<br>• Catchment area<br>• Distance to health facility
School facility surveys	Y1-Y5	Survey CTO	Study area	WASH, school enrollment and attendance rates of children	• MDA conducted at school<br>• Time teacher spend helping with the drug delivery<br>• Teacher trained for school de-worming in past 6 months
Cross-sectional prevalence surveys	Y1, Y4, Y5	Kato-Katz, qPCR	500 (1000 for end-line) individuals per cluster	STH prevalence and intensity, DNA	• Deworming in past year<br>• Deworming source<br>• WASH
Longitudinal prevalence surveys	Y1-Y5	Kato-Katz, qPCR	150 individuals per cluster	STH prevalence, intensity, re-infection, DNA	• Time (minutes) CDD spent at the house on the treatment day<br>• Time spent participate in the treatment day, including time waiting for CDD
cMDA	Y1-Y3	Survey CTO	Intervention clusters	MDA coverage	
Coverage surveys	Y1-Y3	Urine test	50 households per cluster	MDA coverage	• Health workers bring medicines or other treatments by the house (last month)<br>• Health worker left tables for intake later<br>• Number of tablets swallowed<br>• Tablets swallowed while the health worker present<br>• Awareness of drug distribution visit<br>• Change to routine to participate in the treatment<br>• Time (minutes) CDD spent at the house on the treatment day<br>• Time spent participate in the treatment day, including time waiting for CDD

For further details on the study protocol refer to Asbjornsdottir et al PLOS 2018

For peer review only - http://bmjopen.bmj.com/site/about/guidelines.xhtml

Table S2 Activity Table: cMDA-related activities at the India site in the first trial cycle

Nr	Activity	Sub-activity	Trial arm	Implementation	Resource use
1	Drug procurement and supply chain	Procurement	*Both*	*- Drugs for both arms procured centrally by MoH as part of NTD program procurement<br>- DW3 is part of the national bulk purchase of donated drugs through WHO*	*- GSK donating 200,000 doses to the national NTD program for the trial<br>- Annual procurement<br>- Albendazole testing*
		Storage	*Both*	*DW3 trial drugs are stored at central CMC office with A/C (not paid by DW3)*	*Space requirements for 1 round MDA: 1-2 cupboards to keep 25 medium size boxes of tablets (6ft * 4 ft); total space needed for all rounds MDA: 10ft*10ft*
		Transportation	*Both*	*Grants manager travelled to Mumbai to the Government Medical Depot to take the drugs and arrange courier for shipment to DW3 office in Vellore. From Vellore, drugs were taken to sites using DW3 project vehicle.*	*- Grants manager flight to/from Mumbai, per-diems, accommodation<br>- Courier charges Mumbai to Vellore<br>- Fuel Vellore to field sites*
2	Community-wide MDA round 1 *(implemented as mop-up 1 week after SBD)*	Community sensitization	*Int*	*- Implemented right after NDD<br>- Central DW3 team adapts government developed materials to the trial<br>- State health authorities and local social scientists provide input to IEC adaptation through meetings at central level<br>- ASHA FOs and FSs workers organize and run village meetings*	
		Recruitment of CDDs	*Int*	*- 2 days<br>- 164 total CDDs recruited: 32 were already available, 132 were scouted*	*-Staff time:<br>TC: 1 day<br>FM: 2 days<br>FS (11): 4 days<br>FOs (all): 2 days<br>- Transportation allowance: field staff 3Rs/km<br>-Hired vehicle to transport recruiters*
		Training and Piloting	*Int*	*- CDDs 3 days:<br>CDDS/ASHA workers are trained by DW3 Medical Officer at cluster level.<br>- FOs FS 4 days:<br>FOs (109) and FS (11) trained on forms by TC, DM, TL<br>- AE/SAE training for Medical Team 1 day:<br>MO gave training to 2 DW3 study nurses*	*- CDD/ASHAs received travel reimbursement (100 rupees) and refreshments<br>-Travel reimbursement and refreshments for CDD/ASHA workers<br>- No refreshments provided, only regular transport allowance for FOs, FS<br>- No transport allowance for AE/SAE trainings<br>-Fuel for vehicle to transport TC, DM, TL to field (4 days)*
		MDA	*Int*	*- DW3 FS supervise MDA<br>- MDA delivered by CDDs accompanied by FOs, all by foot<br>- 2 MOs and 2 nurses support in the field with AE<br>- 164 CDDs/ASHA workers total, working on average 5 days each for a total of 26 days worked.*	*-108 FOs<br>-1 CDD at a time per FW<br>-Village health nurse hired for 15 days to sensitize, administer and mop-up MDA<br>- CDDs/ASHA workers are paid an honorarium (200 Rs/day) + mobile allowance (50 Rs/round) for a total of 173,400Rs<br>- 44 VHNs , SHNs, CHNs paid 500 Rs/round for a total of 22,000Rs<br>- Office supply costs including printing of logs<br>- Job aids, bags, posters, banners, flip charts*

For peer review only - http://bmjopen.bmj.com/site/about/guidelines.xhtml

		Post-MDA coverage survey	*Int*	*- Includes 1 day of training for FOs by TC and DM<br>- Data processing done centrally by local data team*	*- Staff time: DM,TC,FOs<br>- Office supply costs including printing of logs*
		Mop-up MDA	*Int*	*- FOs and FS did mop-up and a few CDDs/ASHA*	*No allowances given to CDDs/ASHA*
3	Drug procurement and supply chain	Procurement	*Int*	*- Drugs for both arms procured centrally by MoH as part of NTD program procurement<br>- DW3 is part of the national bulk indent of donated drugs through WHO*	*-GSK donating 200,000 doses to the national NTD program for the trial<br>- Albendazole quality control testing*
		Storage	*Int*	*DW3 trial drugs are stored at central CMC office with A/C (not paid by DW3)*	*Space requirements for 1 round MDA: 1-2 cupboards to keep 25 medium size boxes of tablets (6ft * 4 ft); total space needed for all rounds MDA: 10ft*10ft*
		Transportation	*Int*	*Grants Manager travelled to Mumbai to the Government Medical Depot to collect the drugs and arrange courier for shipment to DW3 office in Vellore. From Vellore, drugs were taken to sites using DW3 project vehicle.*	*- Grants Manager flight to/from Mumbai, per-diems, accommodation<br>- Courier charges Mumbai to Vellore<br>- Fuel Vellore to field sites*
4	Community-wide MDA round 2	Community sensitization	*Both*	*- CAB meetings 1 day:<br>TC, PI, FM, MO, IS (4), FS (8), 12 CAB members<br>- Community sensitization meetings 11 days: FM and 3 FS and 5 FO went to 82 villages conducted 82 meetings (5 persons per meeting)<br>- Joined meetings organized by Grama Sabha to sensitize village heads and local community leaders<br>- FS/FO travel by personal bikes*	*- Staff time: PI,TC,MO,FM, FS<br>- Food expense for community-level CAB meetings<br>- BMOs 1000Rs honorarium, others (schoolteachers, block superintendent, opinion leaders etc) 500Rs, Food 237 Rs/person<br>- FS/FOs receive transport allowance 3 Rs per km<br>- Fuel for DW3 project staff (CMC vehicle)*
		Training and Piloting	*Int*	*- CDDs/ASHA 2 days: CDDS/ASHA workers are trained by DW3 MO at cluster level<br>- 4 days: FOs (60) and FS (10) trained on forms by TC, DM, TL.<br>-AE/SAE training for Medical Team 1 day: MO gave training to 2 DW3 study nurses*	*- CDD/ASHAs received travel reimbursement (100 rupees) and refreshments<br>- No refreshments provided, only regular transport allowance for FO, FS trainings<br>- No transport allowance for AE trainings<br>-Fuel for vehicle to transport TC, DM, TL to field (4 days)*
		MDA	*Int*	*-DW3 field supervisors supervise MDA<br>-MDA delivered by CDDs accompanied by FO (70), all on foot<br>- 2 Medical officers and 2 nurses support in the field with adverse event<br>-114 CDDs/ASHA workers total, working on average 5 days each for a total of 12 days worked.*	*- CDDs/ASHA workers are paid an honorarium (200 Rs/day) + mobile allowance (50 Rs/round) for a total of Rs188,650<br>- Office supply costs including printing of logs<br>- Job aids, bags, posters, banners, flip charts*
		Post-MDA coverage survey	*Both*	*- Includes 1 day of training for FOs by TC and DM<br>- Data processing done centrally and by local data team*	*- Staff time: DM,TC, FOs, DM<br>- Printing charges<br>- Office supply costs including printing of logs*
		Mop-up MDA	*Int*	*- FOs and FS did mop-up (and a few CDDs/ASHA)*	*No allowances given to CDDs/ ASHA workers*

Int= Intervention arm; MoH= Ministry of Health; MoE= Ministry of Education; CMC= Christian Medical College, Vellore; TC= trial coordinator; FM= field manager; FS= field supervisor; FO= field officer; DM= data manager

For peer review only - http://bmjopen.bmj.com/site/about/guidelines.xhtml

Table S3 Cost classification of inputs in the DeWorm3 costing tool

No	Input classification	Define	Examples
1	Drugs	Anti-helminthic medication distributed during MDA campaigns and used to treat adults with moderate to high intensity infections	Albendazole
2	Wages and per-diems	Fixed regular payment earned for work or services, per-diem allowances, and any other monetary awards paid to personnel involved in the trial	Per-diems
3	Vehicles and overheads	Means of transportation, including hire and transportation allowance, vehicle maintenance, fuel	Transportation allowance, fuel allowance
4	Equipment and overheads	Supplies and tools that last more than a year and have a unit cost at or above 100 USD; including maintenance and overheads	Mobile phones, Microscope, Equipment maintenance
4	Buildings and overheads	Facilities occupied by DeWorm3 trial and supporting teams	Rent payment, utilities incurred at DeWorm3 site offices
5	Materials and supplies	Commodities required to support an operation or activity	SIM cards, office supplies
6	Other	All other inputs outside of the either of the above categories	Consumables (i.e. food and drinks)

For peer review only

Table S4 Allocation of resource line items to trial activities and categorization of resource line-items into cMDA and research-related and allocation rules of management overheads to trial activities

Resource category	Resource line items	Activity	Activity allocation rule	cMDA	Research
Staff wages and per-diems	Trial PI and core trial staff	Program management	Permanent staff, direct allocation based on % time working on trial	Yes	Yes
	Field staff	Activity	Temporary staff, direct allocation based on number of days supporting activity	Yes	Yes
	Laboratory staff	Prevalence surveys	Permanent staff, direct allocation based on % time working on trial; temporary staff, direct allocation based on number of days supporting activity	No	Yes
	Data staff	Program management	100%	No	Yes
Vehicles and overheads	Vehicles owned	Program management	100%	Yes	Yes
	Vehicles rented	Program management	100%	Yes	Yes
	Transportation related expenses of field and other project staff	Activity	Direct allocation based on number of days supporting activity	Yes	Yes
Equipment and overheads	Office equipment	Program management	100%	Yes	Yes
	Mobile phones	Census	100%	No	Yes
	Field worker kits	Activity	100%	Yes	Yes
	Laboratory equipment	Prevalence survey	100%	No	Yes
Buildings and overheads	Central	Program management	100%	Yes	Yes
	Field offices	Program management	100%	Yes	Yes
Materials and supplies	Stationaries and other materials	Activity	Direct allocation based on number of days supporting activity	Yes	Yes
Other	Trial insurance	Program management	100%	No	Yes

For peer review only - http://bmjopen.bmj.com/site/about/guidelines.xhtml

Table S5 Average annual financial costs of STH cMDA* implementation in India DeWorm3 trial site, USD 2018

Sub-activity	Start-up**		Program management		Drug testing and distribution to sites		CDD recruitment		Community sensitization		Training		cMDA		Coverage survey		Mop-up	
Cost category	US$	%	US$	%	US$	%	US$	%	US$	%	US$	%	US$	%	US$	%	US$	%
Wages and per-diems***	2,027	94.3	26,520	63.4	0	0.0	1,199	57.2	11,283	50.7	4,322	55.1	18,666	66.4	3,232	58.1	2,388	57.8
Buildings and overheads	80	3.7	2,139	5.1	0	0.0	0	0.0	0	0.0	0	0.0	0	0.0	0	0.0	0	0.0
Equipment and overheads	0	0.0	857	2.0	0	0.0	0	0.0	7	0.0	0	0.0	0	0.0	0	0.0	0	0.0
Transportation	22	1.0	7,562	18.1	553	87.0	858	41.0	7,590	34.1	3,360	42.8	7,709	27.4	2,215	39.8	1,665	40.3
Communication	7	0.3	3,378	8.1	0	0.0	0	0.0	0	0.0	0	0.0	205	0.7	0	0.0	0	0.0
Materials and supplies	11	0.5	1,384	3.3	0	0.0	38	1.8	3,078	13.8	135	1.7	395	1.4	120	2.2	75	1.8
Other	2	0.1	0	0.0	83	13.0	0	0.0	309	1.4	26	0.3	1,126	4.0	0	0.0	0	0.0
Total	2,149	100.0	41,840	100.0	636	100.0	2,095	100.0	22,267	100.0	7,843	100.0	28,101	100.0	5,567	100.0	4,128	100.0

*cMDA was implemented as a mop-up following NDD campaign that targeted school-aged children; ** Start-up activities annualized over the duration of the trial (5 years). *** Wages and per-diems include project staff, field workers, and incentives to CDDs. Costs were converted to USD using average annual exchange rate over the study period (1 INR=0.01462 USD) [26]. STH= Soil-Transmitted Helminth infections; cMDA= community-wide Mass Drug Administration; CDD= Community Drug Distributor.

For peer review only

For peer review only - http://bmjopen.bmj.com/site/about/guidelines.xhtml

Table S6 First year financial costs of STH cMDA* implementation in India DeWorm3 trial site, USD 2018: adjusted base rate

	Total (US$)			Average cost Profile (%)	Average cost per person treated per round (US$)	Average cost per Capita per round (US$)
MDA round	1	2	1+2	1+2	1+2	1+2
**TOTAL**	62,819	57,672	120,491	100.0	1.20	0.88
**TOTAL incremental****	4,829	6,577	11,406	9.5	0.11	0.08
Start-up activities***	1,094	1,055	2,149	1.8	0.02	0.02
Program management	21,300	20,540	41,840	34.7	0.42	0.31
Drug testing and distribution to site	304	331	636	0.5	0.01	0.00
CDD recruitment	1,160	1,119	2,279	1.9	0.02	0.02
Community sensitization	13,972	10,235	24,207	20.1	0.24	0.18
CDD bags and job aids	555	428	983	0.8	0.01	0.01
Banners, posters	969	969	1,937	1.6	0.02	0.01
Training	4,826	3,222	8,048	6.7	0.08	0.06
Travel allowance for CDDs	240	170	409	0.3	0.00	0.00
Refreshments for CDDs	10	17	26	0.0	0.00	0.00
cMDA	14,495	16,065	30,560	25.4	0.30	0.22
CDD incentives	2,632	4,577	7,210	6	0.07	0.05
CDD mobile allowance	120	85	205	0.2	0.00	0.00
Coverage survey	3,404	2,803	6,207	5.2	0.06	0.05
Mop-up	2,262	2,302	4,564	3.8	0.05	0.03

*cMDA was implemented as a mop-up following NDD campaign that targeted school-aged children; **Incremental costs represent a subset of rows highlighted in *cursive*, see text for details. *** Start-up activities annualized over the duration of the trial (5 years). Grey shaded rows are a subset of the higher level activity grouping. Total number of people treated in first round of cMDA (cMDA1) was 51’320 (site total population 68’442); in second round (cMDA2) – 49’488 (site total population 68’460); total treated over the two rounds (cMDA1+cMDA2) was 100'808 (total population 136'902). Costs were converted to USD using average annual exchange rate over the study period (1 INR=0.01462 USD) [27]. STH= Soil-Transmitted Helminth infections; cMDA= community-wide Mass Drug Administration; CDD= Community Drug Distributor.

For peer review only - http://bmjopen.bmj.com/site/about/guidelines.xhtml

File 1. DeWorm3 Costing Tool data collection templates

Module1: Planning

A1.1 Planning

	Number of days						
Description	Recruitment	Procurement	Planning trial activities	Developing IEC materials	Developing training materials	Remarks	Source
I	III	IV	V	VI	VII	VIII	IX

A1.2 Pre-trial sensitization

A1.2.1 Pre-trial sensitization meetings

Level of meeting	Location	Type of meeting space	Length of meeting (days)	Number of meetings	Date(s)
National					
State					
District					
Block					
Public health center					
Health sub center					
Cluster					

For peer review only - http://bmjopen.bmj.com/site/about/guidelines.xhtml

A1.2.2 Pre-trial sensitization meetings attendees

	Sensitization meeting							
Description	Level of meeting	Trial staff (Yes/No)	Transportation allowance per meeting (LCU)	Daily lodging allowance (LCU)	Daily food allowance (LCU)	Other allowance (LCU)	Remarks	Source
I	II	III	IV	V	VI	VII	VIII	IX

A1.2.3 Pre-trial sensitization meetings attendees

	Sensitization meeting							
Description	Level of meeting	Trial staff (Yes/No)	Transportation allowance per meeting (LCU)	Daily lodging allowance (LCU)	Daily food allowance (LCU)	Other allowance (LCU)	Remarks	Source
I	II	III	IV	V	VI	VII	VIII	IX

For peer review only - http://bmjopen.bmj.com/site/about/guidelines.xhtml

Module2: Shared resources and program overheads

P2.1 Wages and per-diems

Description	Employed by trial (Yes/No)	Working on trial (%)	Number of staff units (units)	Monthly wage (LCU)	Other monthly benefits (LCU)	Daily per-diem rate (LCU)	Remarks	Source
I	II	III	XVI	XVII	XVIII	XIX	XX	XXI

P2.2 Vehicles and overheads

Description	Purchased by trial (Yes/ No)	Used by trial (%)	Number of vehicles (units)	Make	Year of production	Year of purchase	Price (LCU)	Average annual overhead costs (LCU)	Average annual maintenance costs (LCU)	Remarks	Source
I	II	III	IV	V	VI	VII	VIII	IX	X	XI	XII

For peer review only

For peer review only - http://bmjopen.bmj.com/site/about/guidelines.xhtml

P2.2.1 Reference fuel prices

Fuel	Price units	Price per unit (LCU)	Km per L	L per km	Remarks

P2.2.2 Reference distances

Location	Distance units	Distance (one way)

P2.2.3 Other transportation related expenses

Description	Monthly travel expenditures (LCU)										
	Month, year	Month, year	Month, year	Month, year	Month, year	Month, year	Month, year	Month, year	Month, year	Month, year	Month, year

For peer review only - http://bmjopen.bmj.com/site/about/guidelines.xhtml

P2.3 Equipment and overheads

Description	Purchased by DeWorm3 (Yes/ No)	Used by DeWorm3 (%)	Number of units (units)	Make	Year of production	Year of purchase	Total Price (LCU)	Import duties and other charges (LCU)	Average annual overhead costs (LCU)	Average annual maintenance costs (LCU)	Remarks	Source
I	III	IV	V	VI	VII	VIII	IX	X	XI	XII	XIII	XIV

For peer review only

P2.3.1 Reference office furnishings prices and calculations

Furnishings	Unit price (LCU)	Number of units (units)	Total (LCU)

P2.4 Buildings and overheads

Description	Rented by DeWorm3 (Yes/ No)	Used by DeWorm3 (%)	Number of facilities (units)	Monthly rent per sq foot (LCU)	Size (sq feet)	Average monthly overheads (LCU)	Total including monthly overheads (LCU)	Remarks	Source
I	III	IV	V	VI	VII	VIII	IX	X	XI

For peer review only - http://bmjopen.bmj.com/site/about/guidelines.xhtml

P2.5 Materials and supplies

Description	Number of units (units)	Rate per unit (LCU)	Rate units	Remarks	Source
I	II	III	IV	V	VI

P2.5.1 Other materials and supplies

Description	Monthly materials and supplies expenditures (LCU)										
	Month, year	Month, year	Month, year	Month, year	Month, year	Month, year	Month, year	Month, year	Month, year	Month, year	Month, year

P2.6 Other

Description	Purchased by DeWorm3 (Yes/ No)	Used by DeWorm3 (%)	Number of units (units)	Rate per unit (LCU)	Rate units	Other charges (LCU)	Total Charges (LCU)	Remarks	Source
I	II	III	*IV*	V	VI	VII	VIII	IX	X

For peer review only - http://bmjopen.bmj.com/site/about/guidelines.xhtml

For peer review only

Module3: Activity

A3.1 Sub-activity

Cost classification	Description	Number of units (units)	Units	Insurance (LCU)	Freight (LCU)	Travel charges (LCU)	Handling fee (LCU)	Total (LCU)	Remarks	Source
I	II	III	IV	V	VI	VII	VIII	IX	X	XI

For peer review only

For peer review only - http://bmjopen.bmj.com/site/about/guidelines.xhtml

Stata code for processing data entries collated with the templates to derive intervention costs can be made available on request from authors.

For peer review only

For peer review only - http://bmjopen.bmj.com/site/about/guidelines.xhtml
